# Development of a nomogram for predicting incident heart failure and all-cause mortality in patients with chronic kidney disease: a 3-year follow-up study

**DOI:** 10.3389/fmed.2026.1784717

**Published:** 2026-05-08

**Authors:** Yuxin Jiang, Xiaotong Song, Hongfei Liao, Xinyuan Zhou, Chenyu Sun, Dingxin Zhang, Runzhe Zhou, Xiangjie Yang, Xiaoxia Wang, Dashan Li, Jin Zhang, Yan Li, Ying Wang

**Affiliations:** 1 Department of Nephropathy, The First Affiliated Hospital of Anhui Medical University, Hefei, Anhui, China; 2 Department of Biostatistics and Epidemiology, School of Public Health, Anhui Medical University, Hefei, Anhui, China; 3 Changsha Hospital for Maternal and Child Health Care Affiliated to Hunan Normal University, Changsha, Hunan, China; 4 Department of Pathology, The Affiliated Taizhou People’s Hospital of Nanjing Medical University, Taizhou, Jiangsu, China; 5 Division of Public Health, Infectious Diseases, and Occupational Medicine, Mayo Clinic, Rochester, MN, United States; 6 Mayo Clinic School of Graduate Medical Education, Mayo Clinic College of Medicine and Science, Rochester, MN, United States; 7 School of Public Health, University of Minnesota-Twin Cities, Minneapolis, MN, United States; 8 Department of International Medical, The Second Affiliated Hospital of Anhui Medical University, Hefei, China; 9 Cardiac Imaging Center, The First Affiliated Hospital of Anhui Medical University, Hefei, Anhui, China; 10 Division of Life Sciences and Medicine, Department of Clinical Oncology, The First Affiliated Hospital of USTC, University of Science and Technology of China, Hefei, Anhui, China

**Keywords:** all-cause mortality, chronic kidney disease, incident heart failure, machine learning, nomogram, prediction model

## Abstract

**Background:**

Chronic kidney disease (CKD) confers a substantially elevated risk of cardiovascular (CV) mortality, primarily driven by heart failure (HF) and other CV complications. This study aimed to develop a nomogram for predicting incident HF and all-cause death in patients with CKD, and further identify individuals at high risk of these outcomes.

**Methods:**

We prospectively recruited 440 patients with CKD stages 3–5 and preserved ejection fraction (LVEF) from a nephropathy center between November 2020 to September 2024. Baseline clinical and demographic characteristics, biochemical and echocardiographic parameters, and data from quality-of-life scales were collected. Key predictors were identified using univariate Cox regression, Lasso, Random Forest, and XGBoost algorithms; these identified predictors were then integrated into a nomogram via multivariate Cox regression. Model performance was assessed using the C-index, ROC analysis, and calibration plots.

**Results:**

Over 29 months (IQR 24–35), 104(23.6%) patients met the primary composite endpoints, which included 62(14.1%) incident HF and 42(9.5%) all-cause death. After selecting variables through forward stepwise regression, age[HR 1.030, *P* = 0.016], growth differentiation factor 15(GDF-15) [HR 1.076, *P* < 0.001], lipoprotein(a)[Lp(a)] [HR = 1.011, *P* = 0.002], ferritin(FER) [HR = 1.009, *P* = 0.030], and EuroQol-5D (EQ-5D) score [HR 0.099, *P* = 0.015] were utilized to construct the nomogram. In the training cohort, the model achieved a C-index of 0.772. For temporal validation, time-dependent area under the ROC curve(AUC) values further confirmed its accuracy: 0.779 (95%CI, 0.696–0.863) at 24 months, 0.816(95%CI, 0.743–0.888) at 27 months, 0.836(95%CI, 0.768–0.903) at 32 months, and 0.850(95%CI, 0.771–0.929) at 36 months. Decision curve analysis (DCA) also demonstrated sustained clinical utility across a broad range of threshold probabilities.

**Conclusion:**

This study identified specific predictors of HF and all-cause mortality in CKD patients. This model has enhanced predictive ability for early identification of these risks, thereby facilitating timely clinical interventions to improve the prognosis of CKD patients.

## 1 Introduction

Chronic kidney disease (CKD) has become a critical global public health challenge, with epidemiological data indicating a global prevalence of 15–20% and a persistent upward trend driven by population aging and the epidemic of metabolic syndrome (1, 2). Notably, patients with advanced CKD [glomerular filtration rate (GFR) < 30 mL/min/1.73 m^2^] face a cardiovascular (CV) mortality rating 10∼20 times higher than the general population, with heart failure (HF) and other CV-related deaths accounting for approximately 40∼50% of all mortality in this high-risk population (3, 4). This substantially elevated CV risk is driven by a convergence of traditional and non-traditional risk factors (5). On one hand, traditional risk factors, including hypertension, diabetes, and dyslipidemia, are not only highly prevalent in CKD but also more difficult to control, forming the essential basis for cardiovascular disease (CVD) development. On the other hand, as kidney function declines, a series of unique pathophysiological alterations directly induced by CKD progressively become core drivers of cardiovascular injury. Accumulation of uremic toxins directly impairs vascular endothelial function and triggers sustained oxidative stress. Mineral and bone disorders (CKD-MBD) promote vascular smooth muscle cell osteogenic transdifferentiation, accelerating vascular calcification and myocardial interstitial fibrosis. Chronic microinflammation, characterized by persistently elevated circulating pro-inflammatory cytokines such as interleukin-6 (IL-6) and tumor necrosis factor-alpha (TNF-α), accelerates atherosclerotic plaque formation, destabilization, and adverse myocardial remodeling. Renal anemia leads to inadequate tissue oxygen delivery, compensatory increases in cardiac output, elevated preload, and consequent left ventricular hypertrophy and diastolic dysfunction. Collectively, these CKD-specific mechanisms create a vulnerable myocardial substrate that predisposes to incident HF, particularly heart failure with preserved ejection fraction (HFpEF), even before overt systolic dysfunction develops (5–7).

Traditional cardiovascular risk assessment tools- such as the Framingham Risk Score and the ARIC HF clinical risk prediction equation - exhibit robust predictive performance in the general population, but their discriminative ability is substantially diminished in CKD cohorts (8–11). This diminished predictive accuracy primarily arises from: (1) failure to incorporate kidney-specific indicators such as dynamic GFR fluctuations and proteinuria severity (12, 13); (2) underestimation of mineral and bone metabolism abnormalities in accelerating coronary artery calcification rates (14–16); and (3) neglect of uremia-specific myocardial fibrosis biomarkers and cardiac stress biomarkers [e.g., growth differentiation factor 15 (GDF-15), galectin 3 (Gal-3), soluble ST2 (sST2) and fibroblast growth factor 23 (FGF23)] (17–20). Therefore, there is an urgent need to develop multidimensional prediction tools tailored for CKD patients to improve risk stratification and intervention strategies.

Nomograms, as visual tools for individualized risk prediction, integrate multidimensional biomarkers into an intuitive scoring system and have shown clinical utility in disease diagnosis and mortality risk prediction (21–24). Using a prospective cohort design, this study aimed to develop and validate a nomogram specifically for predicting incident HF and all-cause mortality in patients with CKD stages 3–5 (including those receiving PD and with preserved LVEF). As a result, it can aid clinicians in stratifying risk categories and formulating tailored monitoring plans.

## 2 Materials and methods

### 2.1 Study population

This prospective study, conducted at a tertiary nephrology center (The First Affiliated Hospital of Anhui Medical University) from November 2020 to September 2024, enrolled patients with CKD G3a–G5 (25), with a subset receiving PD therapy. Exclusion criteria included: (1) age < 18 years; (2) current or historical diagnosis of heart failure of any phenotype (including HFrEF, HFmrEF, and HFpEF) based on Framingham criteria and/or echocardiographic evidence; (3) cerebrovascular events (ischemic/hemorrhagic stroke); (4) documented atherosclerotic cardiovascular disease (defined as prior myocardial infarction, coronary revascularization, or angiographically confirmed coronary stenosis ≥ 50%); (5) moderate-to-severe valvulopathy (echocardiographically confirmed stenosis/regurgitation ≥ moderate severity) or prior valve replacement; (6) left ventricular dysfunction (ejection fraction < 50%); (7) peripheral arterial disease (defined as ankle-brachial index < 0.90 or prior lower extremity revascularization); and (8) acute inflammatory states (e.g., peritonitis, autoimmune disorders). A three-tiered screening protocol was rigorously implemented. The initial eligibility assessment involved cross-referencing medical histories, clinical profiles, and laboratory parameters against predefined inclusion criteria. Only fully compliant candidates providing informed consent underwent protocol-mandated echocardiography, culminating in the enrollment of 440 subjects to follow-up for up to 36 months.

This prospective cohort study was approved by the Clinical Research Ethics Committee of the First Affiliated Hospital of Anhui Medical University (Hefei, Anhui, People’s Republic of China; Ethical Approval Number: PJ2020-11-19). All participants provided written informed consent prior to enrollment. The study design, data collection, and follow-up procedures strictly adhere to the ethical principles outlined in the World Medical Association Declaration of Helsinki for research involving human participants.

The current study uses data from the same CKD cohort as our previously published work (PMCID: PMC11728072), which also received approval under the same ethical number (PJ2020-11-19). This approval covers all secondary analyses of the cohort (including supplementary biomarker detection and investigations of different outcomes) and does not require additional ethical review, as confirmed by the above-mentioned Ethics Committee.

### 2.2 Clinical features

Data sources encompassed prospective medical record analysis, and laboratory report to systematically capture demographics, past medical conditions, and medication profiles. Demographic factors included age, gender, smoking status, drinking status, and peritoneal dialysis status. Smoking status was categorized as “ever smoker” (past or current smokers) or “never smoker.” Similarly, alcohol consumption status was classified as “ever alcohol abuse” (past or current alcohol abuse) or “never alcohol abuse.” Physical examination variables included seated systolic blood pressure (SBP), diastolic blood pressure (DBP), heart rate, waist-to-hip circumference ratio (WHR), and body mass index (BMI). Hypertension and diabetes were identified as the comorbidities of interest. The definition of hypertension encompassed two criteria: an average SBP/DBP of ≥ 140/90 mmHg, or the regular administration of antihypertensive drugs. For diabetes, diagnosis relied on self-reported data from participants, including the utilization of glucose-lowering treatments.

### 2.3 Measurements

Peripheral venous blood specimens (10 mL) were obtained from the cubital veins of each participant upon hospital admission, following an overnight fast. Of these specimens, 7 mL of blood and all collected urine samples were dispatched to the Clinical Laboratory of the First Affiliated Hospital of Anhui Medical University. Here, routine blood and urine tests were performed to assess blood chemical profiles and urine biochemical parameters. All results of these routine tests, along with their respective reference ranges, were retrieved from the electronic medical record system, which was updated by the Department of Pathology. Estimated glomerular filtration rate (eGFR) was calculated using the serum creatinine-based equation developed by the Chronic Kidney Disease Epidemiology Collaboration (CKD-EPI) (26). Subsequently, the remaining 3 mL of blood samples were immediately processed via refrigerated centrifugation. The separated serum specimens were then transported on dry ice to the Scientific Research Center of Anhui Medical University, where researchers determined serum concentrations of multiple biomarkers using enzyme-linked immunosorbent assay (ELISA) kits (Elabscience Biotech, Wuhan, China). The detected biomarkers included growth differentiation factor 15 (GDF15), full-length intact fibroblast growth factor 23 (iFGF23), soluble suppression of tumorigenicity 2 (sST2), galectin-3 (GAL3), and α-klotho.

### 2.4 Echocardiography

All echocardiographic assessments were performed using a GE Vivid E95 cardiac ultrasound system. In accordance with the recommendations outlined by the American Society of Echocardiography (ASE) (27, 28), each participant underwent a complete echocardiographic evaluation. This comprehensive assessment incorporated standard transthoracic two-dimensional (2D) imaging, Doppler echocardiographic assessments, and speckle-tracking echocardiography (STE). Left ventricular ejection fraction (LVEF) was determined via the modified Simpson’s biplane technique. For left ventricular mass index (LVMi) calculation, measurements of left ventricular (LV) mass were first acquired using M-mode echocardiography; LV mass was then adjusted for body size using each participant’s body surface area (BSA) to calculate LVMi. Additionally, key diastolic function parameters were collected, including the peak early diastolic transmitral flow velocity (E-wave), the peak early diastolic velocity of the mitral and lateral mitral annuli (e’-wave), and the ratio of the mitral inflow early diastolic velocity to the average e’-wave velocity (E/e’ ratio). For STE, three apical views—apical long-axis, 2-chamber, and 4-chamber views—were employed to quantify longitudinal strain. This parameter was used to evaluate myocardial wall shortening (manifested as negative strain) and lengthening (manifested as positive strain). Global longitudinal strain (GLS) was computed by averaging strain values from the three apical views using standard analysis software. In conventional deformation analysis, shortening is represented by negative values. However, for computational simplicity (and given the absence of positive GLS values in this study), GLS was reported without this negative sign in the present research.

### 2.5 Questionnaires

All patients completed the EuroQol-5D (EQ-5D) questionnaire and the Minnesota Living with Heart Failure Questionnaire (MLHFQ) at baseline assessment. Both instruments are validated tools for assessing quality of life (29–31). The EQ-5D (29, 32) consists of the visual analogue scale (VAS) and incorporates five health domains: mobility, self-care, usual daily activities, pain/discomfort, and anxiety/depression. Each dimension is measured on a 5-level ordinal scale: no problems, slight problems, moderate problems, severe problems, and extreme problems or inability to perform (specifically for mobility, self-care, and usual activities). The EQ-5D health utility index is theoretically anchored at 0 (representing the health state “dead”) and 1 (denoting perfect health) by the EuroQol Group’s valuation algorithms. In this study, the EQ-5D questionnaire was administered only at baseline, and all participants completed the questionnaire independently. The MLHFQ (30, 31) incorporates 21 items using a 6-point (0–5) Likert response format, with total scores ranging from 0 to 105, where higher values indicate worse quality of life. This validated instrument consists of two subscales: a physical domain (8 items, 0–40 points) and an emotional domain (5 items, 0–25 points).

### 2.6 Outcomes

Potential HF symptoms were identified during follow-up. The process started with structured telephone interviews and questionnaires, followed by symptom surveillance questionnaires, clinical visits, and repeated echocardiography. Based on the Framingham heart failure diagnostic criteria (adapted for telephone administration), the following symptoms were assessed: (1) dyspnea at rest or with exertion; (2) orthopnea (number of pillows used or inability to lie flat); (3) paroxysmal nocturnal dyspnea; (4) peripheral edema (location and degree); (5) fatigue or reduced exercise tolerance; (6) new or worsening nocturnal cough; (7) palpitations or rapid heartbeat; (8) history of acute severe dyspnea requiring emergency care; and (9) rapid weight gain ( > 2 kg in 3 days). Symptom severity was documented using standardized questionnaires, and positive findings prompted clinical evaluation with repeat echocardiography. All-cause hospitalization and treatment records were retrieved for all patients, while monitoring data were concurrently collected via questionnaires and clinical follow-ups. For patients with incomplete data, supplementary telephone interviews were conducted with family members; patients with unavailable complete data were excluded. New-onset HF diagnoses were adjudicated by a consensus panel comprising three cardiologists based on the Framingham Heart Failure Diagnostic Criteria (11), requiring independent review of all available documentation. The primary endpoint was incident HF and all-cause mortality.

### 2.7 Statistical analysis

In this prospective cohort study, the missing data rate of all variables were below 5%. Missing values were imputed using the Random Forest (RF) method from the Scikit-learn library (Python, version 1.1.3)—a choice justified by its superior performance in handling complex non-linear data structures and preserving variable relationships relative to traditional methods (e.g., mean imputation, multiple imputation). The normality of data distribution was assessed via the Shapiro-Wilk test. Descriptive statistics were expressed as mean ± standard deviation (SD), and *t*-tests were employed for group comparisons when dealing with variables that conformed to a normal distribution. Variables with non-normal distributions were summarized as median and interquartile range (IQR), and analyzed by Mann-Whitney U tests. Categorical variables were characterized by frequencies (n, %) and evaluated for group differences using chi-square tests.

To identify potential biomarkers, a multi-stage analytical framework was applied to the complete dataset. First, univariate Cox proportional hazards regression was used for initial screening. Subsequently, three machine learning approaches were implemented: LASSO regression for dimensionality reduction and feature optimization, and two ensemble learning algorithms, RF and Extreme Gradient Boosting (XGBoost), to extract the top ten most significant predictive features. Hyperparameters for all machine learning models were optimized using 10-fold cross-validation with grid search, minimizing the cross-validated partial likelihood deviance for the LASSO model and maximizing the area under the receiver operating characteristic curve (AUROC) for RF and XGBoost. Specifically, in the LASSO analysis, 10-fold cross-validation and 1,000 iterations were set, with L1 regularization for variable selection and importance analysis. For the RF model, a systematic grid search was performed over the following hyperparameter space: n_estimators [50, 100, 200, 500], max_depth [None, 10, 20, 30], min_samples_split [2, 5, 10], and min_samples_leaf [1, 2, 4]. The final selected parameters were n_estimators = 100, max_depth = None, min_samples_split = 2, and min_samples_leaf = 1. The Friedman mean squared error (mse) was used as the node splitting criterion. For the XGBoost model, a grid search was conducted over: n_estimators [50, 100, 200], learning_rate [0.01, 0.05, 0.1, 0.2], max_depth [3, 6, 8, 10], min_child_weight [1, 3, 5, 6], subsample [0.6, 0.8, 1.0], and lambda [0, 1, 5, 10, 15]. The final selected parameters were n_estimators = 100, learning_rate = 0.1, max_depth = 8, min_child_weight = 6, subsample = 0.8, and lambda = 1. The objective function was set to reg: squared-error based on the squared error loss function for regression tasks.

Candidate variables were identified by intersecting the results of the Cox univariate analysis with those from each of the three machine learning methods using Venn diagrams, a comprehensive approach chosen to prioritize variables with consistent predictive value across diverse analytical frameworks. Collinearity among these candidate variables was assessed using variance inflation factors (VIF), with values below 5 indicating no substantial multicollinearity. Subsequently, these candidates were then incorporated into the Cox regression model with bidirectional stepwise method to determine independent prognostic factors. For this fitted model, the proportional hazards assumption was tested using Schoenfeld residuals (function cox.zph in R), with a global *P* > 0.05 and individual variable *P* > 0.05 considered to indicate no violation. The identified independent predictors were integrated into the nomogram prediction model using the “rms” package (version 6.2.0). Due to the limited number of endpoint events in CKD G3 and G4 stages, formal stratified analyses for individual CKD stages were not performed. Instead, we performed clinically meaningful stratified analyses by non-dialysis dependent CKD (G3+G4) vs. ESKD (G5), as well as sensitivity analyses to verify model robustness.

The dataset was randomly partitioned into a training set (*n* = 308) and a validation set (*n* = 132) in a 7:3 ratio. The training set was used for nomogram development and internal validation, while the validation set was reserved for model evaluation. The concordance index (C-index) derived from internal validation via the Bootstrap method (with 1,000 resamples) was utilized to evaluate the accuracy of the nomogram. To account for the time factor, time-dependent receiver operating characteristic (ROC) curve analysis was conducted using “timeROC” package (version 0.4) and the “pROC” package (version 1.18.5), with “month” as as the time unit and default cut-off points (0.2, 0.4, 0.6, 0.8), supplemented by additional analyses at clinically relevant thresholds (e.g., 0.1, 0.5, 0.9) to ensure robustness of predictive performance. Decision curve analysis (DCA) was performed to assess the model’s clinical utility. We determined the optimal risk score cut-off value using the R package “maxstat” (version 0.7–25) with subgroup size constraints (min > 25%, max < 75%), stratified patients into high- and low-risk groups accordingly, and subsequently assessed prognostic differences between these groups by Kaplan-Meier survival analysis with Log Rank testing.

To evaluate the incremental predictive value of each individual predictor, we compared the full model with reduced models that excluded one predictor at a time. The improvement in model fit was assessed using the likelihood ratio test (LRT), with the change in chi-square (**Δ*χ^2^***) and corresponding P value calculated for each comparison. The change in discrimination performance was quantified by the difference in the concordance index (ΔC-index) between the full model and each reduced model, and its statistical significance was evaluated using 1,000 bootstrap resamples to obtain 95% confidence intervals and *P*-values. Additionally, time-dependent net reclassification improvement (NRI) and integrated discrimination improvement (IDI) were computed. The NRI measures the net proportion of subjects correctly reclassified into higher or lower risk categories, while the IDI quantifies the overall improvement in the average predicted risk difference between events and non-events. All analyses were performed using R software with the survival, rms, and survIDINRI packages. The results are summarized in Supplementary Tables S5a,b. All statistical analyses were executed using R version 4.2.3 and Python version 3.11.4, and a *P* -< 0.05 was regarded as statistically significant.

## 3 Results

### 3.1 Baseline descriptive characteristics

**FIGURE 1:**
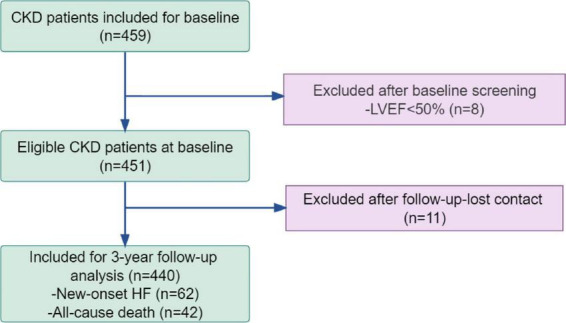
Enrollment flowchart: baseline population, exclusions, and analyzed subject.

The participant recruitment process is detailed in Figure 1. Following a median follow-up of 29 months [interquartile range (IQR), 24–35 months], 11 participants (2.5% of the cohort) withdrew from the study. Among the 440 participants who completed follow-up, 104 (23.6%) experienced a composite primary endpoint event, including 62 (14.1%) cases of incident HF and 12(2.7%) cardiovascular (CV) death, and 30 (6.8%) non-cardiovascular deaths. The cumulative numbers of composite events at 24, 27, 32, and 36 months were 30 (6.8%), 42 (9.5%), 66 (15.0%), and 86 (19.5%), respectively. The event rates varied by CKD stage, as detailed in Supplementary Table S1: 7.4% in stage G3, 35.7% in stage G4, and 25.1% in stage G5, consistent with the overall rate of 23.6%. Baseline characteristics of the cohort are summarized in Table 1. The study population had a mean age of 52.44 ± 11.97 years, and 60.91% were female. The median estimated glomerular filtration rate (eGFR_*CDK*–*EPI*_) was 6.00 (4.00, 11.00) mL/min/1.73 m^2^. Peritoneal dialysis (PD) was administered to 57.27% of participants, and among 252 PD patients, the median treatment duration was 26.00 (IQR 7.00, 54.00) months, with all using continuous ambulatory peritoneal dialysis (CAPD). Notably, when compared with the no-event group, patients who experienced HF or death were significantly older (age 57.42 ± 11.19 vs. 50.90 ± 11.78 years, *P* < 0.001), and had higher circulating levels of GDF15 (2249.74 ± 670.90 vs. 1933.69 ± 686.80 pg/mL, *P* = 0.0040), and E/e’ ratio [11.70 (9.63,14.17) vs. 10.76 (8.65,13.01), *P* = 0.034]. Conversely, hemoglobin (HGB) levels were significantly lower in the event group (93.94 ± 17.27 vs. 101.04 ± 19.79 g/L, *P* = 0.022).

**TABLE 1:** Comparisons of baseline clinical characteristics, lab/echo indicators, and quality of life scales (MLHFQ and EQ-5D) between the event group and non-event group.

Variables	All (*n* = 440)	Event group (*n* = 104)	Non-event group (*n* = 336)	χ^2^/t	*p*-value
Clinical characteristics					
Age (yrs)	52.44 ± 11.97	57.42 ± 11.19	50.90 ± 11.78	−3.514	**< 0.001**
Female gender (%)	268(60.91)	60(57.70)	208(61.91)	0.296	0.586
WHR	0.95 (0.90, 0.99)	0.98 (0.91, 1.00)	0.95 (0.90, 0.98)	−1.773	0.076
Systolic BP, mm Hg	145.23 ± 21.34	145.56 ± 24.07	145.13 ± 20.42	−0.127	0.899
Diastolic BP, mm Hg	90.00 ± 12.76	87.27 ± 13.03	90.85 ± 12.56	1.77	0.078
Heart rate, beats/min	81.46 ± 13.69	81.35 ± 11.94	81.50 ± 14.18	0.071	0.944
BMI, kg/m^2^	22.61 ± 3.31	22.51 ± 3.42	22.64 ± 3.27	0.26	0.795
Hypertension (%)	356 (80.91)	84 (80.77)	272 (80.95)	0.001	0.977
Smoking status, ever (%)	94 (21.36)	22 (21.15)	72 (21.43)	0.002	0.966
Drinking status, ever (%)	104 (23.64)	24 (23.08)	80 (23.81)	0.012	0.913
Diabetes (%)	76 (17.27)	26 (25.00)	50 (14.88)	2.846	0.092
Peritoneal dialysis (%)	252 (57.27)	60(57.69)	192 (57.14)	0.005	0.944
Complete blood count (with normal range)					
RBC (4.35–5.80 × 10^12^/L)	3.23 (2.83, 3.73)	3.18 (2.70, 3.64)	3.24 (2.83, 3.74)	0.735	0.463
HGB (130–175 g/L)	99.36 ± 19.46	93.94 ± 17.27	101.04 ± 19.79	2.315	**0.022**
WBC (3.50–9.50 × 10^9^/L)	6.20 (4.94, 7.64)	6.51 (4.84, 7.25)	6.19 (4.95, 7.95)	0.702	0.484
PLT (125–350 × 10^9^/L)	172.00 (132.00, 206.00)	172.00 (131.00, 199.00)	172.00 (132.00, 209.00)	0.193	0.848
NEUT (1.80–6.30 × 10^9^/L)	4.17 (3.15, 5.42)	3.97 (2.98, 5.13)	4.20 (3.24, 5.66)	1.09	0.276
LYMPH (1.10–3.20 × 10^9^/L)	1.26 (0.95, 1.57)	1.29 (0.96, 1.57)	1.24 (0.95, 1.57)	0.346	0.730
EO (0.02–0.52 × 10^9^/L)	0.15 (0.08, 0.23)	0.17 (0.09, 0.21)	0.15 (0.08, 0.24)	−0.006	0.996
Biochemical parameters (with normal range)					
eGFR ( > 90 mL/min/1.73 m^2^)	6.00 (4.00, 11.00)	6.00 (4.00, 9.00)	6.00 (4.00, 12.00)	0.936	0.346
BUN (3.10–8.00 mmol/L)	19.08 ± 7.42	19.67 ± 6.73	18.90 ± 7.61	−0.652	0.515
CRE (57–97 μmol/L)	726.66 ± 395.69	746.14 ± 358.74	720.59 ± 406.33	−0.405	0.686
UA(155–357 μmol/L)	429.67 ± 92.87	414.75 ± 89.43	434.32 ± 93.43	1.326	0.186
TBIL (0–23 μmol/L)	8.02 (6.71, 10.15)	8.56 (6.65, 10.15)	7.97 (6.80, 10.15)	−0.59	0.556
IBIL (0–19 μmol/L)	6.86 (5.65, 8.68)	7.06 (5.58, 8.81)	6.83 (5.66, 8.68)	−0.584	0.560
Lp(a) (0–300 mg/L)	327.71 ± 280.83	393.31 ± 331.34	307.40 ± 259.91	−1.699	0.094
Ca (2.11–2.52 mmol/L)	2.23 (2.10, 2.37)	2.23 (2.02, 2.32)	2.23 (2.12, 2.38)	1.294	0.196
P (0.85–1.51 mmol/L)	1.59 (1.29, 1.90)	1.58 (1.36, 1.83)	1.60 (1.27, 1.90)	−0.317	0.752
Fe (7.80–32.20 μmol/L)	12.07 ± 4.49	12.10 ± 4.53	12.06 ± 4.48	−0.052	0.958
FER (10–120 μg/L)	277.81 ± 255.92	328.77 ± 318.56	262.04 ± 230.88	−1.389	0.169
ALP (35–100 U/L)	102.45 ± 111.94	106.31 ± 92.61	101.25 ± 117.27	−0.281	0.779
CRP (0–10.00 mg/L)	2.01 (0.69, 7.47)	2.52 (0.96, 6.20)	1.81 (0.67, 7.47)	−0.991	0.322
GDF15 (pg/mL)	2008.39 ± 696.15	2249.74 ± 670.90	1933.69 ± 686.80	−2.902	**0.004**
FGF23 (pg/mL)	259.29 ± 278.79	243.61 ± 242.53	264.14 ± 288.91	0.462	0.644
sST2 (ng/mL)	42.73 ± 9.37	42.58 ± 9.63	42.79 ± 9.26	0.122	0.903
GAL3 (ng/mL)	8.71 ± 1.80	8.96 ± 1.65	8.61 ± 1.85	−1.032	0.304
NT-proBNP ( < 450 pg/mL)	390.63 ± 115.83	406.86 ± 125.25	384.33 ± 111.32	−1.041	0.300
α-Klotho (237.25–432.75 pg/mL)	1280.34 ± 231.66	1298.05 ± 210.98	1273.46 ± 238.86	−0.566	0.572
CK-MB (0–24 U/L)	12.47 ± 3.76	13.26 ± 4.55	12.23 ± 3.44	−1.492	0.140
Echocardiography					
LVEF (%)	62.00 (59.00, 65.00)	62.00 (58.00, 64.00)	62.00 (59.00, 65.00)	1.168	0.242
GLS (%)	16.00 (14.00, 18.10)	15.90 (14.10, 18.70)	16.10 (14.10, 17.90)	0.339	0.737
LVMi (g/m^2^)	113.94 ± 26.45	116.79 ± 25.40	113.06 ± 26.70	−0.886	0.377
E/e’ratio	11.14 (8.91, 13.45)	11.70 (9.63, 14.17)	10.76 (8.65, 13.01)	−2.119	**0.034**
Questionnaires					
MLHFQ (0–105)	16.11 ± 16.02	19.44 ± 17.14	15.07 ± 15.52	−1.722	0.087
EQ-5D (0–1)	0.94 (0.87, 1.00)	0.92 (0.82, 1.00)	0.94 (0.88, 1.00)	1.400	0.153

BMI, body mass index; WHR, waist-to-hip circumference ratio; BP, blood pressure; RBC, red blood cell count; HGB, hemoglobin; WBC, white blood cell count; PLT, platelet; NEUT, neutrophils; LYMPH, lymphocytes; EO, eosinophils; eGFR, estimated glomerular filtration rate; BUN, blood urea nitrogen; CRE, creatinine; UA, uric acid; TBIL, total bilirubin; IBIL, indirect bilirubin; Lp(a), lipoprotein(a); Ca, calcium; P, phosphorus; Fe, iron; FER, ferritin; ALP, alkaline phosphatase; CRP, C-reactive protein; GDF15, growth differentiation factor 15; FGF23, fibroblast growth factor 23; sST2, soluble suppression of tumorigenicity 2; GAL3, galectin-3; NT-proBNP, N-terminal prohormone brain natriuretic peptide; CK-MB, creatine kinase-MB isoenzyme; LVEF, left ventricular ejection fraction; E/e’ ratio, mitral early diastolic inflow velocity (E-wave, m/s) to early diastolic mitral annular velocity (e’, m/s) ratio; LVMi, left ventricular mass index; GLS, global longitudinal strain; MLHFQ, Minnesota Living with Heart Failure Questionnaire score; EQ-5D, EuroQol-5D score. Bold values indicate statistical significance (*P* < 0.05).

After random allocation of samples, 308 patients were assigned to the training set and 132 to the validation set. The median eGFR in the training group was 6.00 mL/min/1.73 m^2^ (IQR 4.00–11.00), while in the validation group it was 6.00 mL/min/1.73 m^2^ (IQR 4.00–12.00). No significant differences were observed in baseline clinical characteristics, laboratory/echocardiographic indicators, or quality of life scales between the training and validation groups (Table 2). In the training group, 72 patients (23.38%) progressed to composite outcome, compared with 32 patients (24.24%) in the validation group.

**TABLE 2:** Comparison of baseline clinical characteristics, lab/echo indicators, and quality of life scales (MLHFQ and EQ-5D) between the Training cohort and Validation cohort.

Variables	All (*n* = 440)	Training cohort (*n* = 308)	Validation cohort (*n* = 132)	*p*-value
Clinical characteristics				
Age (yrs)	52.44 ± 11.97	52.61 ± 11.77	52.04 ± 12.42	0.645
Female gender (%)	268(60.91)	189(61.36)	79(59.85)	0.848
WHR	0.95 (0.90, 0.99)	0.95(0.90,0.99)	0.95(0.90,0.99)	0.614
Systolic BP, mm Hg	145.23 ± 21.34	145.31 ± 21.10	145.03 ± 21.91	0.899
Diastolic BP, mm Hg	90.00 ± 12.76	89.756 ± 12.537	90.568 ± 13.261	0.542
Heart rate, beats/min	81.46 ± 13.69	81.35 ± 13.90	81.73 ± 13.17	0.792
BMI, kg/m^2^	22.61 ± 3.31	22.76 ± 3.30	22.26 ± 3.30	0.143
Hypertension (%)	356 (80.91)	255(82.79)	101(76.52)	0.161
Smoking status, ever (%)	94 (21.36)	66(21.43)	28(21.21)	0.960
Drinking status, ever (%)	104 (23.64)	57(18.51)	19(14.39)	0.240
Diabetes (%)	76 (17.27)	57(18.51)	19(14.39)	0.296
Peritoneal dialysis (%)	252 (57.27)	176(57.14)	76(57.58)	0.933
Complete blood count (with normal range)				
RBC (4.35–5.80, × 10^12^/L)	3.23 (2.83, 3.73)	3.23 (2.83,3.75)	3.24(2.82,3.73)	0.987
HGB (130–175, g/L)	99.36 ± 19.46	100.10 ± 19.80	97.64 ± 18.53	0.226
WBC (3.50–9.50, × 10^9^/L)	6.20 (4.94, 7.64)	6.19 (4.95,7.62)	6.40 (4.84,7.68)	0.685
PLT (125–350, × 10^9^/L)	172.00 (132.00, 206.00)	169.00 (132.00, 205.00)	180.00 (132.00, 209.00)	0.403
NEUT (1.80–6.30, × 10^9^/L)	4.17 (3.15, 5.42)	4.17 (3.15,5.43)	4.19 (3.22,5.42)	0.991
LYMPH (1.10–3.20, × 10^9^/L)	1.26 (0.95, 1.57)	1.26 (0.96,1.54)	1.21 (0.94,1.68)	0.947
EO (0.02–0.52, × 10^9^/L)	0.15 (0.08, 0.23)	0.16 (0.09,0.24)	0.14 (0.08,0.22)	0.241
Biochemical parameters (with normal range)				
eGFR ( > 90, mL/min/1.73 m^2^)	6.00 (4.00, 11.00)	6.00(4.00,11.00)	6.00(4.00,9.25)	0.537
BUN (3.10–8.00, mmol/L)	19.08 ± 7.42	19.23 ± 7.58	18.75 ± 7.01	0.542
CRE (57–97, μmol/L)	726.66 ± 395.69	727.52 ± 398.60	724.66 ± 388.86	0.945
UA(155–357,μmol/L)	429.67 ± 92.87	431.77 ± 91.34	424.83 ± 96.14	0.474
TBIL (0–23, μmol/L)	8.02 (6.71, 10.15)	8.01(6.86,10.14)	8.08(6.38,10.19)	0.748
IBIL (0–19, μmol/L)	6.86 (5.65, 8.68)	6.87(5.79,8.68)	6.83(5.39,8.74)	0.684
Lp(a) (0–300, mg/L)	327.71 ± 280.83	331.36 ± 297.88	324.90 ± 259.26	0.834
Ca (2.11–2.52, mmol/L)	2.23 (2.10, 2.37)	2.23(2.12,2.38)	2.20(2.09,2.33)	0.201
P (0.85–1.51, mmol/L)	1.59 (1.29, 1.90)	1.59(1.29,1.89)	1.59(1.31,1.90)	0.771
Fe (7.80–32.20, μmol/L)	12.07 ± 4.49	12.03 ± 4.34	12.16 ± 4.84	0.784
FER (10–120, μg/L)	277.81 ± 255.92	270.64 ± 264.71	278.99 ± 260.44	0.769
ALP (35–100, U/L)	102.45 ± 111.94	331.36 ± 297.88	324.90 ± 259.26	0.834
CRP (0–10.00, mg/L)	2.01 (0.69, 7.47)	1.94(0.67,6.72)	2.52(0.78,8.06)	0.255
GDF15 (pg/mL)	2008.39 ± 696.15	1979.64 ± 684.39	2075.48 ± 718.39	0.187
FGF23 (pg/mL)	259.29 ± 278.79	261.32 ± 281.94	254.54 ± 271.22	0.816
sST2 (ng/mL)	42.73 ± 9.37	42.07 ± 9.50	44.32 ± 8.83	0.065
GAL3 (ng/mL)	8.71 ± 1.80	8.77 ± 1.87	8.57 ± 1.62	0.416
NT-proBNP ( < 450 pg/mL)	390.63 ± 115.83	388.82 ± 117.27	394.98 ± 112.15	0.684
α–Klotho (237.25–432.75 pg/mL)	1280.34 ± 231.66	1271.36 ± 244.72	1301.93 ± 195.06	0.311
CK-MB (0–24 U/L)	12.47 ± 3.76	12.63 ± 3.81	12.11 ± 3.60	0.191
Echocardiography				
LVEF (%)	62.00 (59.00, 65.00)	62.00 (59.00,65.00)	62.00 (60.00,65.00)	0.162
GLS (%)	16.00(14.00, 18.10)	16.25 (14.10,18.15)	15.65 (14.00,17.78)	0.452
LVMi (g/m^2^)	113.94 ± 26.45	115.32 ± 28.06	110.72 ± 21.89	0.095
E/e’ratio	11.14 (8.91, 13.45)	62.00 (59.00,65.00)	62.00 (60.00,65.00)	0.454
Questionnaires				
MLHFQ (0–105)	16.11 ± 16.02	15.19 ± 14.39	18.03 ± 18.85	0.221
EQ-5D (0–1)	0.94 (0.87, 1.00)	0.94(0.88,1.00)	0.94(0.84,1.00)	0.388
Outcome				
Incident HF and all-cause mortality (n, %)	104 (23.64)	72 (23.38)	32 (24.24)	0.941

BMI, body mass index; WHR, waist-to-hip circumference ratio; BP, blood pressure; RBC, red blood cell count; HGB, hemoglobin; WBC, white blood cell count; NEUT, neutrophils; PLT, platelet; LYMPH, lymphocytes; EO, eosinophils; eGFR, estimated glomerular filtration rate; BUN, blood urea nitrogen; CRE, creatinine; UA, uric acid; TBIL, total bilirubin; IBIL, indirect bilirubin; Lp(a), lipoprotein(a); Ca, calcium; P, phosphorus; Fe, iron; FER, ferritin; ALP, alkaline phosphatase; CRP, C-reactive protein; GDF15, growth differentiation factor 15; FGF23, fibroblast growth factor 23; sST2, soluble suppression of tumorigenicity 2; GAL3, galectin-3; NT-proBNP, N-terminal prohormone brain natriuretic peptide; CK-MB, creatine kinase-MB isoenzyme; LVEF, left ventricular ejection fraction; E/e’ ratio, mitral early diastolic inflow velocity (E-wave, m/s) to early diastolic mitral annular velocity (e’, m/s) ratio; LVMi, left ventricular mass index; GLS, global longitudinal strain; MLHFQ, Minnesota Living with Heart Failure Questionnaire score; EQ-5D, EuroQol-5D score; HF, heart failure.

### 3.2 Screening for potential biomarkers

**FIGURE 2:**
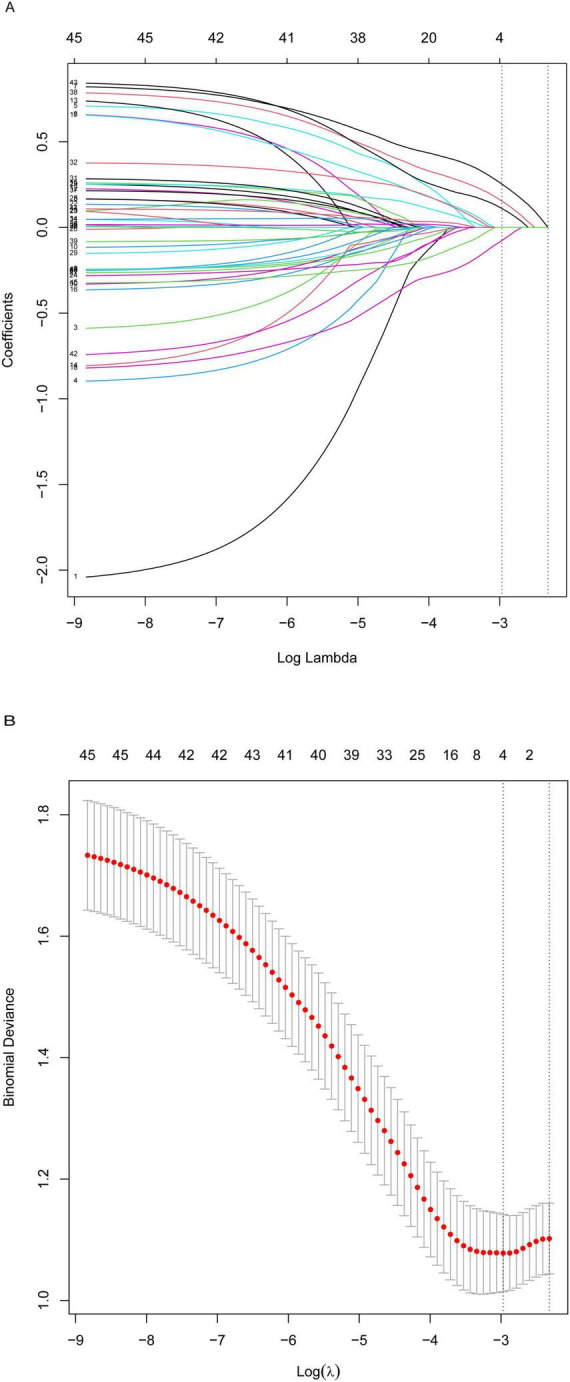
LASSO regression regularization path and lambda optimization: **(A)** coefficient trajectories during shrinkage and **(B)** cross-validation tuning for optimal lambda.

Univariate Cox analysis initially identified eight potential predictors: age, total bilirubin (TBIL), indirect bilirubin (IBIL), lipoprotein(a) [Lp(a)], ferritin (FER), GDF15, MLHFQ, and EQ-5D (Table 3). Subsequently, LASSO regression was applied for feature dimensionality reduction. The coefficient path plot (Figure 2A) illustrates the variable shrinkage process, and ten-fold cross-validation determine regularization parameter (λ = 0.098) when the cross-validation error reached its minimum (Figure 2B). Ultimately, four key biomarkers were selected: age, HGB, GDF15 and E/e’.

**TABLE 3:** Univariate Cox proportional hazards regression analysis for composite endponit.

Variables	HR	95%CI	*P*-value
Clinical characteristics			
Age (yrs)	1.042	1.015–1.069	**0.002**
Female gender (%)	0.835	0.477–1.460	0.526
WHR	6.776	0.260–176.812	0.250
Systolic BP, mm Hg	1.001	0.988–1.014	0.876
Diastolic BP, mm Hg	0.985	0.964–1.007	0.184
Heart rate, beats/min	0.994	0.974–1.015	0.564
BMI, kg/m^2^	0.990	0.909–1.077	0.808
Hypertension (%)	0.884	0.441–1.774	0.729
Smoking status, ever (%)	1.099	0.562–2.148	0.783
Drinking status, ever (%)	1.104	0.576–2.115	0.766
Diabetes (%)	1.463	0.780–2.742	0.235
Peritoneal dialysis (%)	1.008	0.581–1.751	0.976
Complete blood count (with normal range)			
RBC (4.35–5.80, × 10^12^/L)	1.016	0.946–1.091	0.663
HGB (130–175, g/L)	0.989	0.974–1.005	0.168
WBC (3.50–9.50, × 10^9^/L)	0.889	0.773–1.022	0.098
PLT (125–350, × 10^9^/L)	1.001	0.996–1.005	0.770
NEUT (1.80–6.30, × 10^9^/L)	0.905	0.782–1.047	0.179
LYMPH (1.10–3.20, × 10^9^/L)	0.795	0.430–1.468	0.464
EO (0.02–0.52, × 10^9^/L)	0.731	0.096–5.561	0.762
Biochemical parameters (with normal range)			
eGFR ( > 90, mL/min/1.73 m^2^)	0.984	0.961–1.007	0.171
BUN (3.10–8.00, mmol/L)	1.008	0.970–1.047	0.691
CRE (57–97, μmol/L)	1.000	0.999–1.001	1.000
UA(155–357,μmol/L)	0.979	0.739–1.296	0.881
TBIL (0–23, μmol/L)	1.076	1.008–1.149	**0.028**
IBIL (0–19, μmol/L)	1.089	1.016–1.167	**0.015**
Lp(a) (0–300, mg/L)	1.001	1.000–1.002	**0.006**
Ca (2.11–2.52, mmol/L)	0.536	0.163–1.765	0.305
P (0.85–1.51, mmol/L)	1.044	0.533–2.044	0.900
Fe (7.80–32.20, μmol/L)	1.001	0.940–1.067	0.970
FER (10–120, μg/L)	1.001	1.000–1.002	**0.040**
ALP (35–100, U/L)	1.001	0.999–1.003	0.506
CRP (0–10.00, mg/L)	1.003	0.993–1.013	0.561
GDF15 (pg/mL)	1.001	1.000–1.001	**< 0.001**
FGF23 (pg/mL)	1.000	0.999–1.001	0.639
sST2 (ng/mL)	0.996	0.963–1.029	0.797
GAL3 (ng/mL)	1.084	0.906–1.296	0.379
NT-proBNP ( < 450 pg/mL)	1.001	0.999–1.004	0.324
α–Klotho (237.25–432.75 pg/mL)	1.000	0.999–1.002	0.634
CK-MB (0–24 U/L)	1.061	0.989–1.139	0.096
Echocardiography			
LVEF (%)	0.969	0.927–1.014	0.177
GLS (%)	0.971	0.874–1.080	0.591
LVMi (g/m^2^)	0.998	0.989–1.007	0.676
E/e’ratio	1.032	0.945–1.127	0.478
Questionnaires			
MLHFQ (0–105)	1.018	1.001–1.035	**0.035**
EQ-5D (0–1)	0.148	0.035–0.638	**0.010**

BMI, body mass index; WHR, waist-to-hip circumference ratio; BP, blood pressure; RBC, red blood cell count; HGB, hemoglobin; WBC, white blood cell count; PLT, platelet; NEUT, neutrophils; LYMPH, lymphocytes; EO, eosinophils; eGFR, estimated glomerular filtration rate; BUN, blood urea nitrogen; CRE, creatinine; UA, uric acid; TBIL, total bilirubin; IBIL, indirect bilirubin; Lp(a), lipoprotein(a); Ca, calcium; P, phosphorus; Fe, iron; FER, ferritin; ALP, alkaline phosphatase; CRP, C-reactive protein; GDF15, growth differentiation factor 15; FGF23, fibroblast growth factor 23; sST2, soluble suppression of tumorigenicity 2; GAL3, galectin-3; NT-proBNP, N-terminal prohormone brain natriuretic peptide; CK-MB, creatine kinase-MB isoenzyme; LVEF, left ventricular ejection fraction; E/e’ ratio, mitral early diastolic inflow velocity (E-wave, m/s) to early diastolic mitral annular velocity (e’, m/s) ratio; LVMi, left ventricular mass index; GLS, global longitudinal strain; MLHFQ, Minnesota Living with Heart Failure Questionnaire score; EQ-5D, EuroQol-5D score. Bold values indicate statistical significance (*P* < 0.05).

**FIGURE 3:**
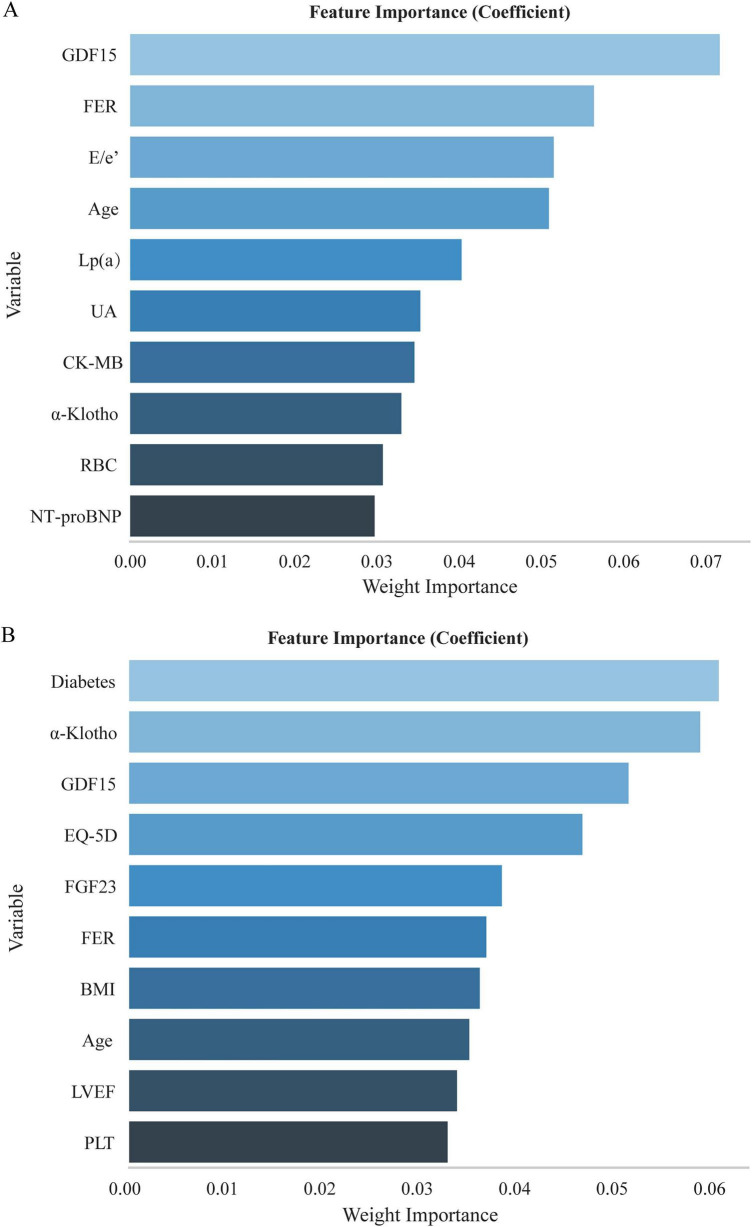
Variable importance ranking from **(A)** random forest algorithm and **(B)** XGBoost classifier models.

In the RF model, the Friedman mean squared error (friedman_mse) served as the splitting criterion, with 100 trees (n_estimators = 100), no depth restriction, and a minimum purity gain of 0.0. Variable importance ranking revealed GDF15, FER, E/e’, age, Lp(a), uric acid (UA), creatine kinase-MB (CK-MB), α-klotho, red blood cell count (RBC), N-terminal pro-B-type natriuretic peptide (NT-proBNP) as top predictors (Figure 3A). Meanwhile, the XGBoost model utilized an objective function of reg: squarederror, a learning rate of 0.1 to control gradient updates, and a dual regularization framework with max_depth = 8, lambda = 1, and min_child_weight = 6 to constrain tree growth. The important ranking here featured diabetes mellites, α-klotho, GDF15, EQ-5D score, FGF23, FER, BMI, age, LVEF, PLT (Figure 3B).

**FIGURE 4:**
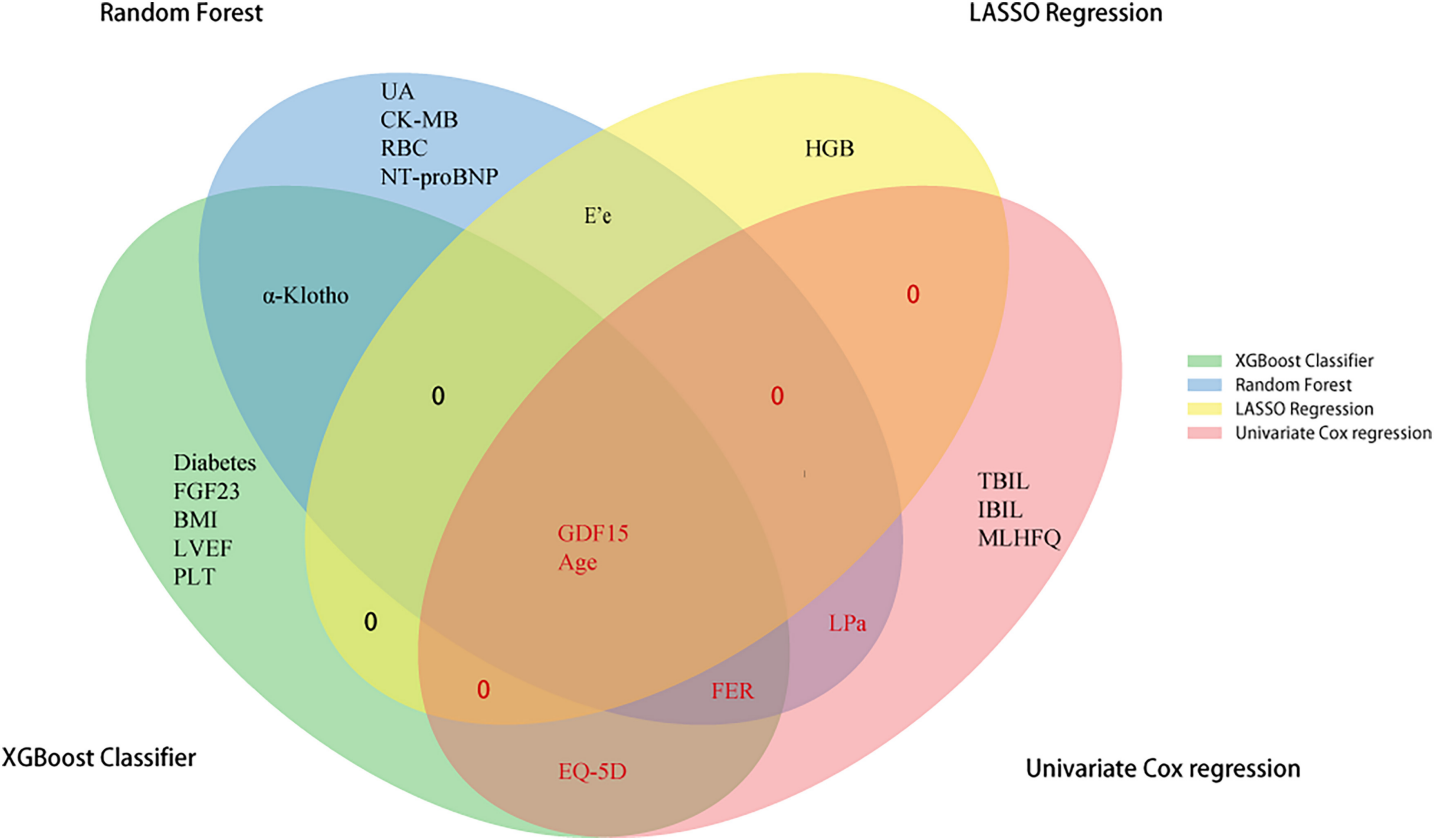
Venn diagram of biomarkers identified by LASSO regression, random forest, and XGBoost models. Red indicates variables selected by union of intersections with univariate Cox.

Notably, a Venn diagram analysis identified five intersection variables—age, GDF15, FER, EQ-5D score, Lp(a)—as candidate biomarkers by overlapping results from univariate Cox analysis and the three machine learning methods (Figure 4).

Collinearity among these five predictors was assessed using VIF. All VIF values were below 2.5 (Supplementary Table S2), indicating no substantial multicollinearity.

### 3.3 Multivariate Cox regression analysis

A Cox proportional hazards model was used to evaluate the impact of potential biomarkers on composite outcome. Bidirectional stepwise regression (direction = “both”) was applied to select variables, accounting for multiple factors influencing survival time. The Cox regression model showed the following five predictors were significantly and independently associated with composite adverse endpoint. Age was associated with a hazard ratio (HR) of 1.030 [95% confidence interval (CI): 1.006–1.056, *P* = 0.016], meaning each 1-year increase in age was associated with a 3.0% higher risk of the composite endpoint. For GDF15, an HR of 1.076 (95% CI: 1.037–1.118, *P* < 0.001), showed that a 100 pg/mL increase in its level was associated with a 7.6% higher risk. Lp(a), had an HR of 1.011 (95% CI: 1.004–1.018, *P* = 0.002), meaning a 10 mg/L elevation in concentration corresponded to a 1.1% risk increment. Similarly, FER exhibited an HR of 1.009 (95% CI: 1.001–1.017, *P* = 0.030), showing that a 10 μg/L increase was linked to a 0.9% higher risk. Conversely, the EQ-5D scores had an HR of 0.099 (95% CI: 0.013–0.755, *P* = 0.015), suggesting that a 0.1 - unit improvement in this score was associated with a 9.0% reduction in the risk of composite events (Table 4). We tested the proportional hazards assumption for the final Cox model using Schoenfeld residuals. All individual predictors satisfied the assumption (all *P* > 0.05), and the global test was also non-significant (χ^2^ = 7.03, P = 0.218). The detailed results were: Age (χ^2^ = 2.78, *P* = 0.099), Lp(a) (χ^2^ = 2.94, *P* = 0.086), FER (χ^2^ = 0.84, *P* = 0.376), GDF15 (χ^2^ = 1.48, *P* = 0.235), and EQ-5D (χ^2^ = 0.001, *P* = 0.954). The Schoenfeld residual plots (Supplementary Figure S1) showed stable patterns over time, further supporting the proportional hazards assumption.

**TABLE 4:** Multivariate Cox proportional hazards regression for predicting composite endpoints.

Predictor	Estimate	SE	*Z*	*p*	Hazard ratio	Lower	Upper
GDF15	0.074	0.019	3.825	< 0.001	1.076	1.037	1.118
FER	0.009	0.004	2.174	0.030	1.009	1.001	1.017
EQ-5D	−2.31	1.035	−2.231	0.026	0.099	0.013	0.755
Lp(a)	0.011	0.003	3.151	0.002	1.011	1.004	1.018
Age	0.030	0.012	2.405	0.016	1.030	1.006	1.056

GDF15, growth differentiation factor 15; FER, ferritin; EQ-5D, EuroQol-5D score; Lp(a), lipoprotein(a).

We further evaluated the model’s performance in two clinically relevant subgroups: non-dialysis dependent CKD (NDD-CKD, stages G3+G4) and ESKD (stage G5). The nomogram maintained consistent predictive performance in both strata, with C-indices of 0.870 (95% CI 0.738–1.001) in the NDD-CKD subgroup and 0.747 (95% CI 0.694–0.801) in the ESKD subgroup. To assess whether the predictive value of the five core predictors is independent of CKD stage, we added CKD stage (as a categorical variable) as an additional covariate in the original multivariate Cox regression model (Model 2, Supplementary Table S3). After adjustment for CKD stage, all five predictors remained independently and significantly associated with the composite endpoint, with hazard ratios and *P*-values similar to those in the original model (Model 1). These results confirm that the predictive value of our core predictors is independent of CKD staging, demonstrating the robustness of the model’s core conclusions.

### 3.4 Development of nomogram

**FIGURE 5:**
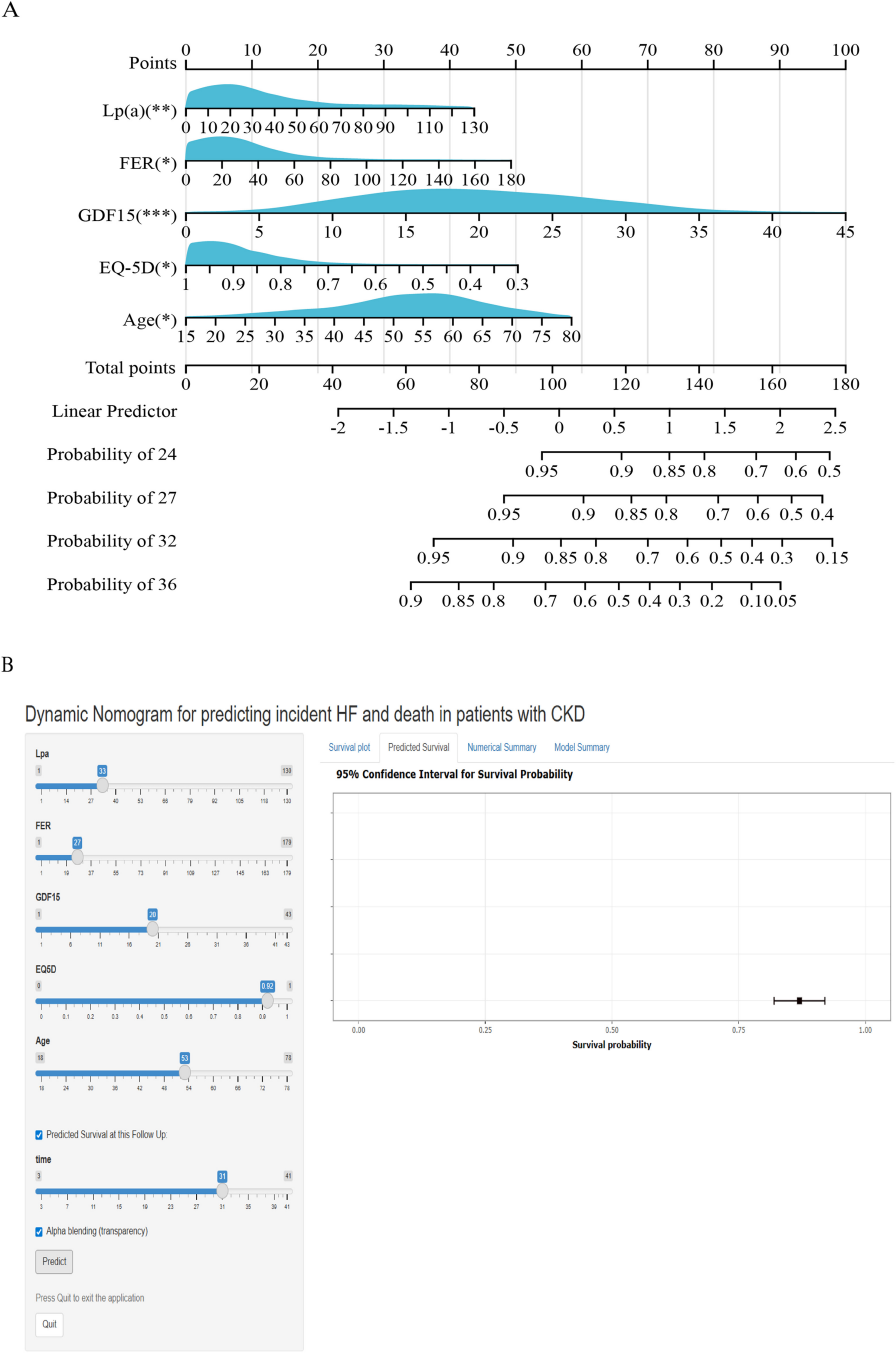
Nomogram for predicting time-dependent composite endpoints in CKD patients. **(A)** Nomogram construction incorporating five independent predictors: Age, Lp(a), FER, GDF15, and EQ-5D score. *P < 0.05, **P < 0.01, ***P < 0.001. **(B)** Interactive online version available at https://jiang221.shinyapps.io/dynnomapp.

A nomogram model was constructed using the four variables identified from multivariate Cox regression, with individual scores assigned based on regression coefficients (Figure 5A). An online dynamic version of the nomogram is accessible at https://jiang221.shinyapps.io/dynnomapp (Figure 5B).

### 3.5 Internal validation

Internal validation via the Bootstrap method (1,000 resamples) yielded a concordance index (C-index) of 0.772 (95% CI 0.725–0.819), demonstrating the model’s significant predictive capability for composite outcomes. Notably, as patient age increased, GDF15, FER, and Lp(a) levels rose, and EQ-5D scores decreased, the cumulative nomogram scores increased, and the predicted risk of the composite outcome rose accordingly.

**FIGURE 6:**
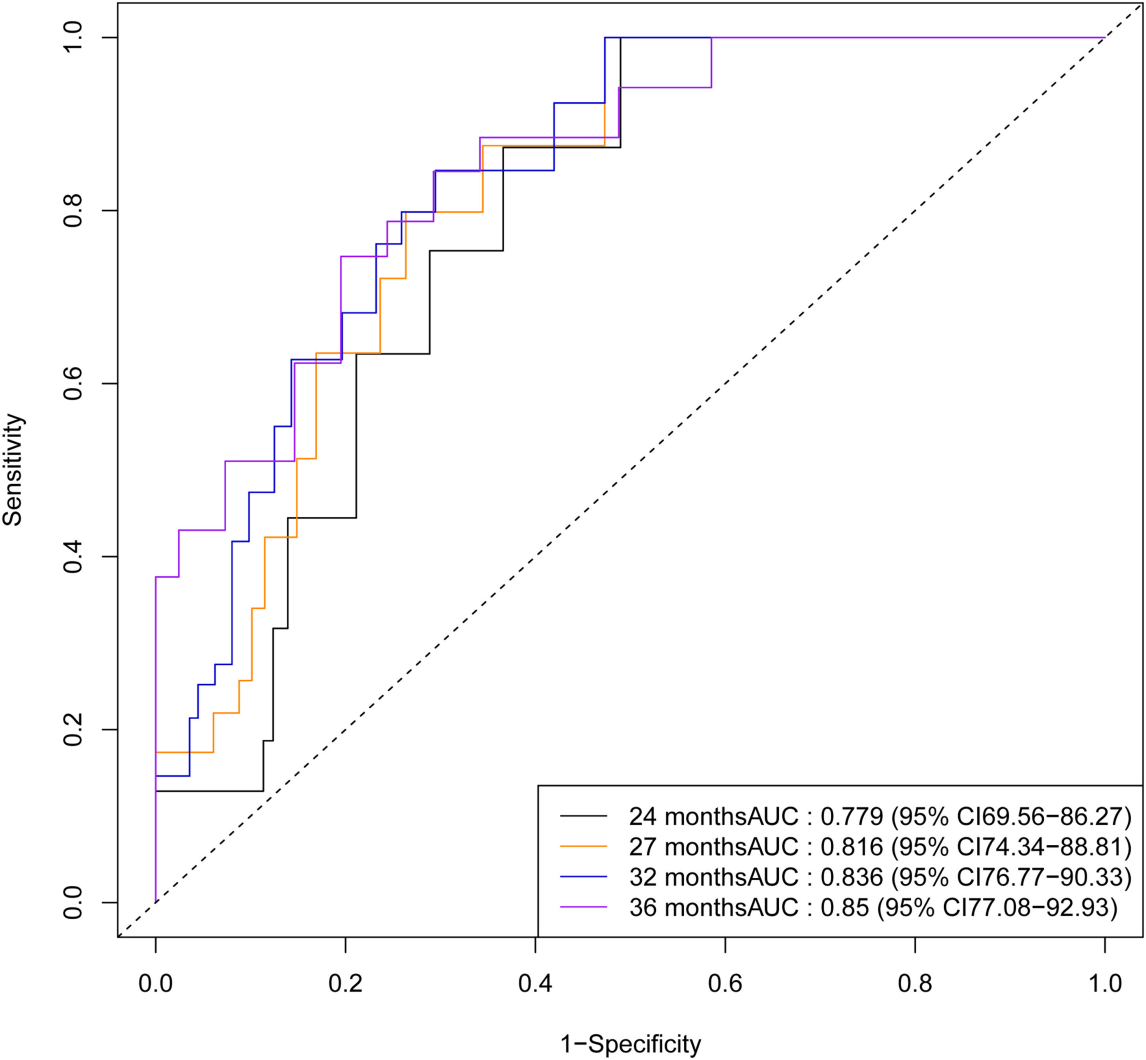
Time-dependent receiver operating characteristic curves of training cohort at specified time quantiles.

**FIGURE 7:**
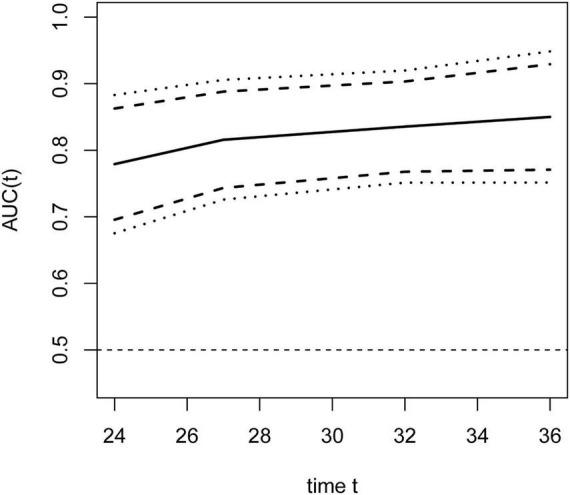
Area under the curve (AUC) values of the training cohort for time-dependent roc curves across time quantiles.

To evaluate the temporal impact on prediction, time-dependent ROC analyses (Figure 6) were performed at prespecified time points of 24, 27, 32, and 36 months. Earlier time points (e.g., 12 months) were not analyzed due to an insufficient number of events, which would have yielded unreliable estimates with wide confidence intervals. The dynamic AUC(t) curves (Figure 7) showed a steady increase from 0.779 (95% CI 0.696–0.863) at 24 months to 0.816 (95% CI, 0.743–0.888) at 27 months, 0.836 (95% CI, 0.768–0.903) at 32 months, and finally 0.850 (95% CI 0.771–0.929) at 36 months.

**FIGURE 8:**
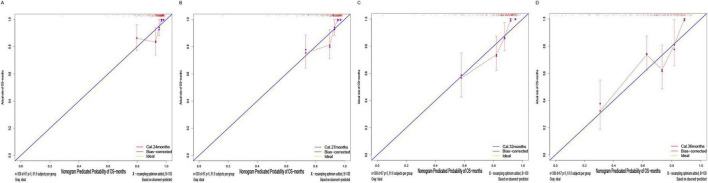
Calibration plots of the training cohort for internal validation of the nomogram at prespecified time points (24/27/32/36 months): **(A)** 24 months; **(B)** 27 months; **(C)** 32 months; **(D)** 36 months.

**FIGURE 9:**
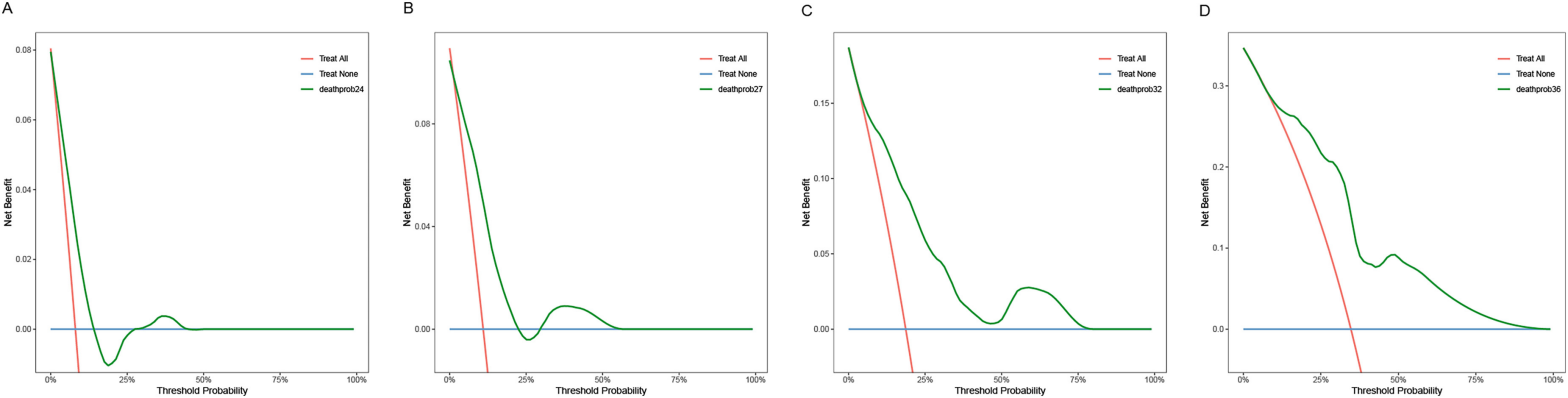
Decision curve analysis of the training cohort nomogram at prespecified time points (24/27/32/36 months): **(A)** 24 months; **(B)** 27 months; **(C)** 32 months; **(D)** 36 months.

The calibration plots (Figure 8) showed overall consistent agreement between predicted and observed composite event risks at the four time points. Quantitative assessment using Brier scores and calibration slopes (Supplementary Table S4) further supported these observations. Meanwhile, decision curve analysis (DCA; Figure 9) showed that the model’s clinical utility became more robust as the threshold probability ranges expanded over time, validating its robustness in predicting composite outcomes within the CKD population.

**FIGURE 10:**
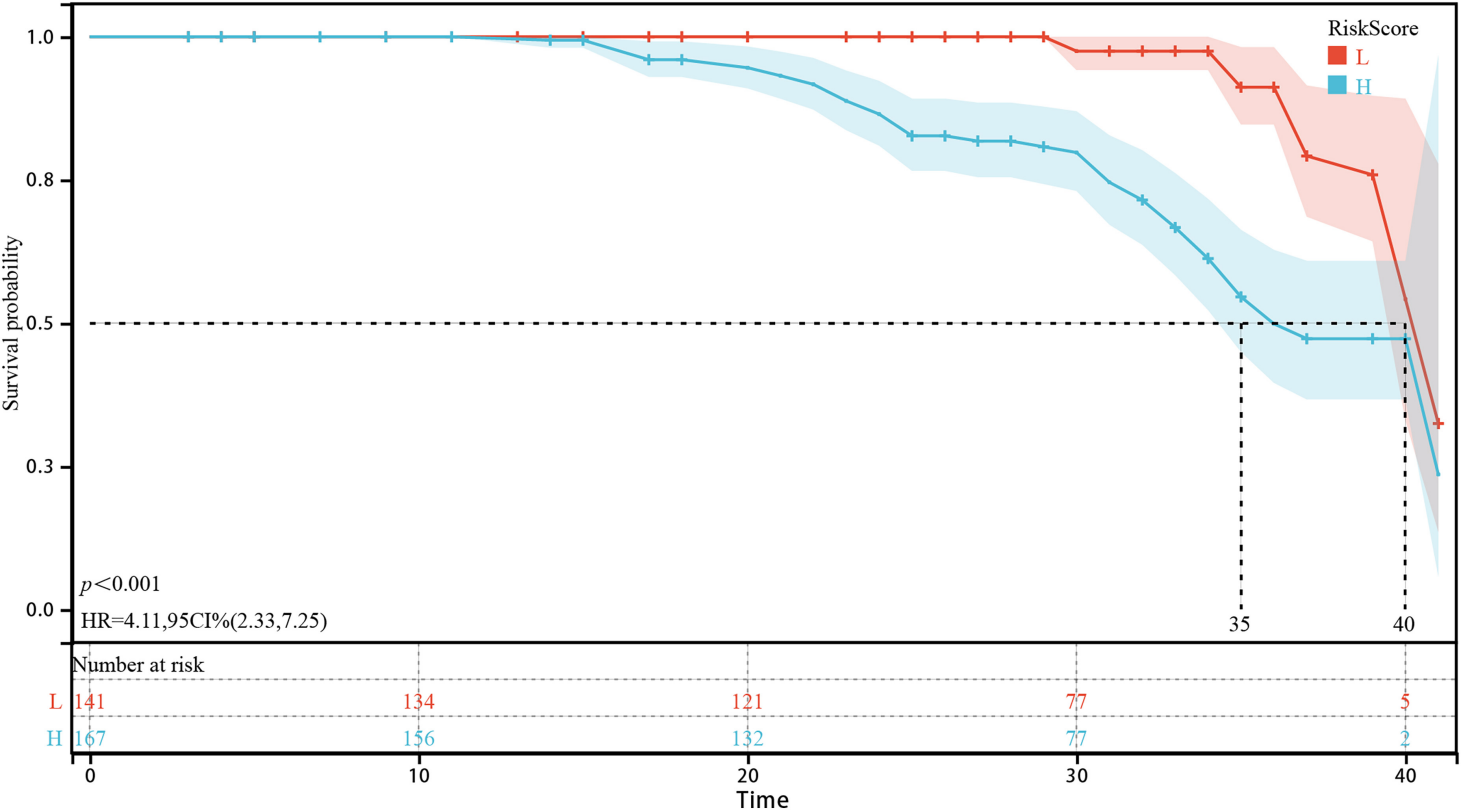
Kaplan-Meier curves of the training cohort for overall survival of CKD patients stratified by risk score.

Using the optimal RiskScore cut-off value of −0.163, patients were stratified into high- and low-risk groups, revealing a statistically significant difference in prognosis between the two groups via Log Rank test (*p* < 0.001; Figure 10).

### 3.6 External validation

**FIGURE 11:**
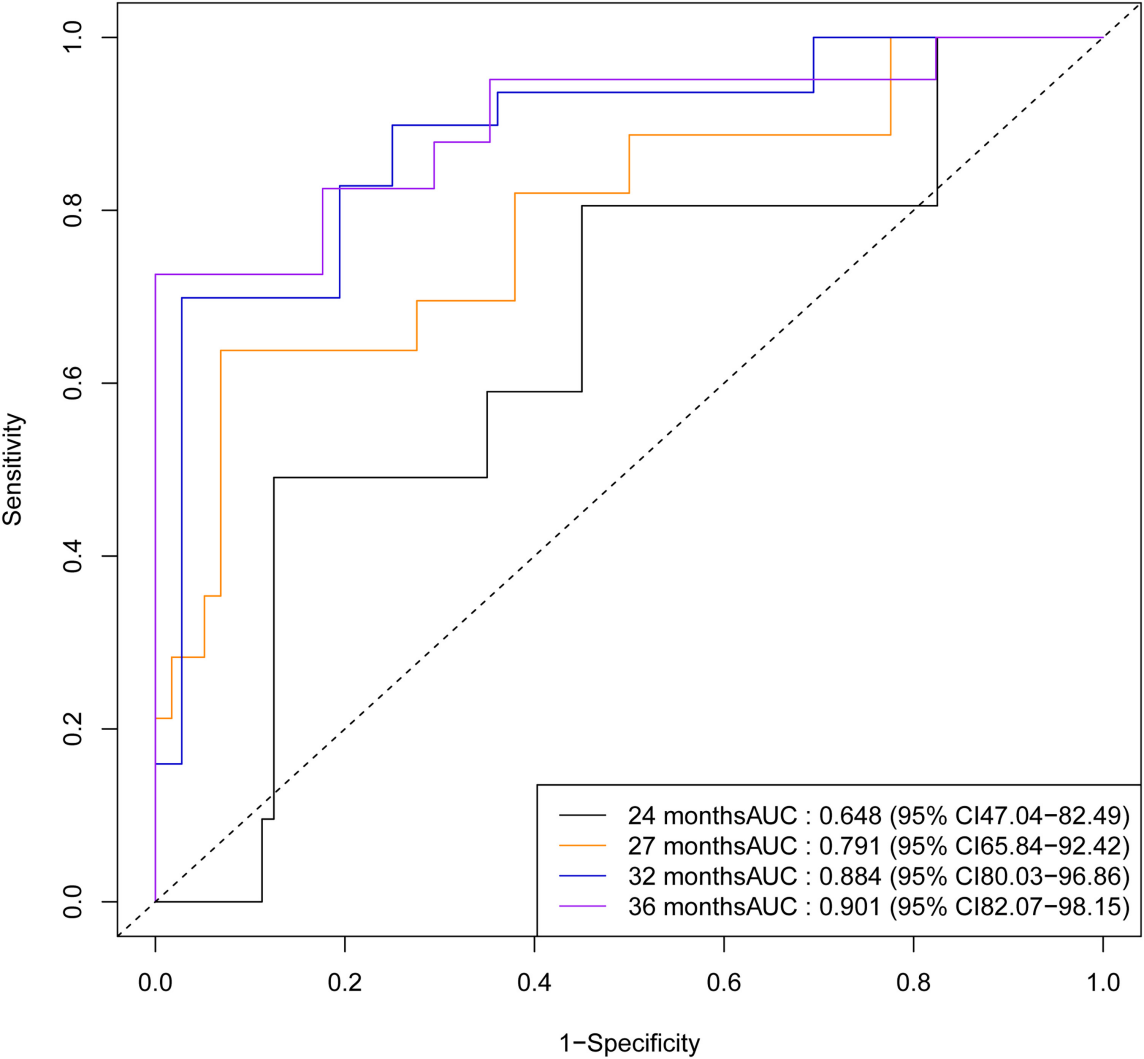
Time-dependent receiver operating characteristic curves of validation cohort at specified time quantiles.

**FIGURE 12:**
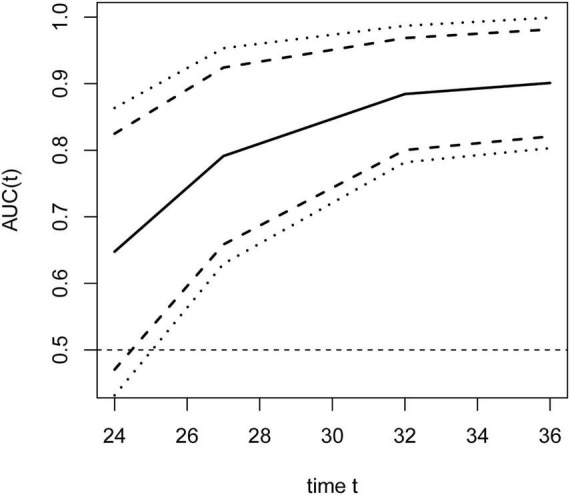
Area under the curve (AUC) values of the validation cohort for time-dependent ROC curves across time quantiles.

In the validation set, trends in ROC curves (Figure 11) and AUC values (Figure 12) were consistent with those in the training cohort: AUC values showed a steady upward trend, increasing from 0.648 (95% CI 0.470–0.825) at 24 months to 0.791 (95% CI 0.658–0.924) at 27 months, further increasing to 0.884 (95% CI 0.800–0.969) at 32 months, and finally reaching 0.901 (95% CI 0.821–0.982) by 36 months.

**FIGURE 13:**
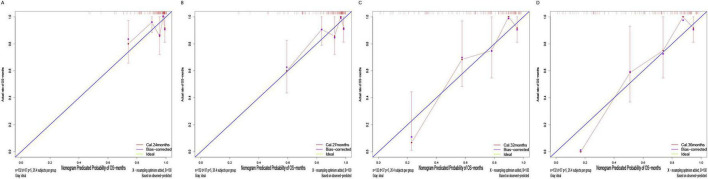
Calibration plots of the validation cohort for internal validation of the nomogram at prespecified time points (24/27/32/36 months): **(A)** 24 months; **(B)** 27 months; **(C)** 32 months; **(D)** 36 months.

**FIGURE 14:**
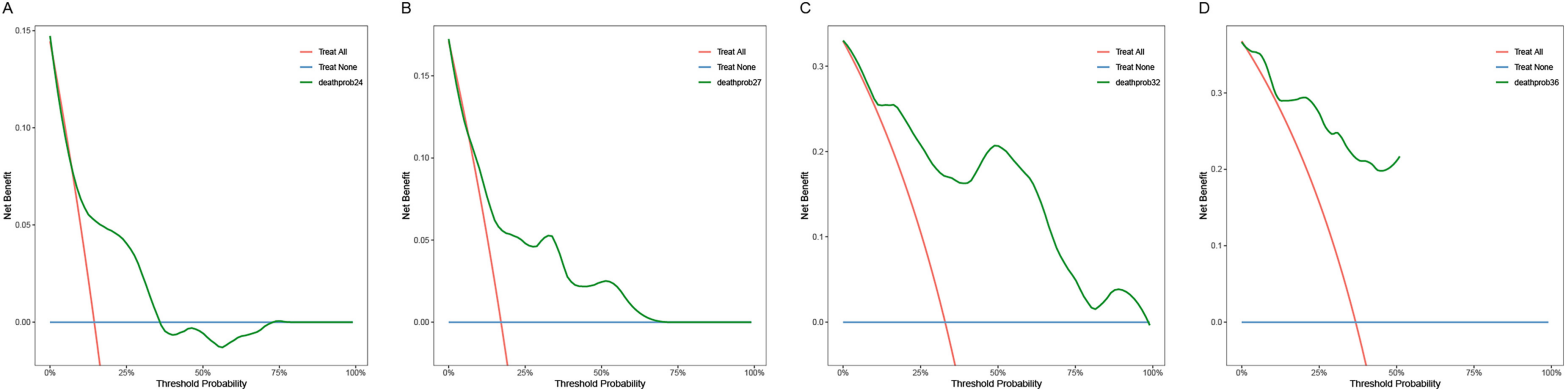
Decision curve analysis of the verification cohort nomogram at prespecified time points (24/27/32/36 months): **(A)** 24 months; **(B)** 27 months; **(C)** 32 months; **(D)** 36 months.

Similarly, the calibration curve plot (Figure 13) demonstrated overall consistency between predicted and observed values. The Brier scores and calibration slopes for the validation cohort are also provided in Supplementary Table S4. DCA (Figure 14) indicates that the clinical utility of this model becomes more robust as the range of threshold probabilities expands over time, thereby validating its robustness in predicting composite outcomes in the CKD population.

## 4 Discussion

This study developed a dynamic time-dependent nomogram based on a Cox regression model, integrating patient-reported quality-of-life metrics (EQ-5D scores), the novel biomarker circulating GDF15, and clinical parameters [FER, Lp(a), age] to predict the risks of incident HF and all-cause death in patients with CKD. This model exhibited robust discriminative ability (C-index: 0.768) and time-stratified predictive accuracy, with AUC values of 0.806, 0.848, and 0.913 at follow-up time points of 27, 32, and 35 months, respectively. Significantly, the inclusion of EQ-5D scores in cardiovascular risk stratification challenges traditional assessment frameworks reliant solely on laboratory markers, underscoring the value of multidimensional health evaluation in patients with advanced CKD. Furthermore, the nomogram demonstrated superior predictive accuracy compared to standalone biomarkers like Lp(a) and FER, highlighting the clinical utility of integrating diverse predictors into a unified decision tool for personalized risk stratification.

The time-dependent AUC trajectory—rom 0.686 at 22 months to 0.913 at 35 months—reflects the dynamic progression of cardiovascular pathology in CKD, which aligns with the “time-varying risk hypothesis”; uremic toxins and metabolic disturbances gradually exacerbate myocardial injury (33). The identified inflection point at 32 months may correspond to the clinical threshold for overt cardiovascular decompensation, providing a rationale for stratified surveillance strategies.

The identified biomarkers have revealed unique molecular mechanisms and pathological processes involved in the deterioration of cardiovascular function. GDF15, identified as an important risk predictor in our study, was associated with a 7.6% increase in the risk of composite endpoints for every 100 pg/mL elevation in patients with CKD. Current evidence suggests that GDF15 may participate in the pathogenesis of CVD in CKD patients through the following mechanisms. GDF-15 is a member of the TGF-β superfamily and a stress inducible cytokine. Under physiological conditions, GDF-15 is expressed at low levels in tissues such as the placenta, prostate, kidney, liver, and colon (17), but its expression is significantly upregulated under pathological conditions including inflammation, hypoxia, ischemia, and myocardial injury (34). In CKD patients, accumulation of uremic toxins induces systemic chronic inflammation, leading to elevated levels of inflammatory cytokines such as TNF-α, IL-1β, and M-CSF, which directly promote GDF15 upregulation (35). Concurrently, declining kidney function reduces GDF15 clearance and increases its production, with serum GDF15 levels showing a significant negative correlation with eGFR (36, 37). These CKD-specific mechanisms collectively result in persistently elevated GDF15, which then directly participates in the development and progression of cardiovascular disease. Experimental evidence indicates that GDF15 exacerbates atherosclerotic plaque formation by promoting IL-6-dependent inflammatory responses and upregulates caspase-3 to induce apoptosis and autophagy, thereby destabilizing plaques (38). Conversely, GDF15 deficiency improves plaque stability by attenuating CCR2-dependent macrophage chemotaxis and enhancing collagen deposition (39), further supporting the pro-inflammatory and pro-apoptotic role of GDF15 in CKD-related atherosclerosis. In our nomogram, GDF15 provided the largest incremental predictive value among all five predictors: the likelihood ratio test showed a Δχ^2^ of 14.701 (*P* < 0.001) and the C-index increased by 0.074 (95% CI 0.011–0.136, *P* < 0.001) after adding GDF15 (Supplementary Table S5a). Time-dependent NRI and IDI increased progressively from 24 to 36 months (e.g., NRI at 36 months: 0.451 [95% CI 0.208–0.659], IDI: 0.140 [95% CI 0.050–0.265]), indicating that its predictive power strengthens with longer follow-up (Supplementary Table S5b). It is precisely this broad regulatory capacity over cardiovascular pathological processes that enables GDF15 to predict cardiovascular risk in CKD patients independently of traditional risk factors. In our study, adding GDF15 to the prediction model significantly improved the predictive ability for all-cause mortality and HF events, further validating its clinical value in risk stratification of CKD patients.

Our study reveals that Lp(a) is an important risk predictor for cardiovascular events in patients with CKD, with its level significantly positively correlated with the occurrence of the composite endpoint. In CKD patients, decreased renal clearance, protein loss, and compensatory hepatic synthesis lead to markedly elevated Lp(a) levels, causing its persistent accumulation in the circulation (40, 41). The unique microinflammatory state and oxidative stress in CKD enhance the interaction between oxidized phospholipids on the Lp(a) surface and the TLR4/CD36/NF-κB pathway, exacerbating vascular inflammation, monocyte adhesion, and smooth muscle cell proliferation (42, 43), thereby promoting the formation of unstable plaques. Concurrently, fibrinolytic dysfunction is common in CKD patients, and the high homology of Apo(a) with plasminogen enables competitive inhibition of plasminogen activation, increasing thrombotic risk, which is particularly prominent in dialysis patients (44). Regarding vascular calcification, oxidized phospholipids carried by Lp(a) induce the osteogenic transdifferentiation of vascular smooth muscle cells, accelerating aortic valve and coronary artery calcification, a process significantly amplified in the context of CKD-mineral and bone disorder (CKD-MBD) (45). Multiple cohort studies have confirmed the independent predictive value of Lp(a) for CVD in CKD patients. The Chronic Renal Insufficiency Cohort (CRIC) study (46) followed 3,939 CKD patients for a median of 7.5 years and showed that compared with the lowest Lp(a) quartile ( < 9.8 mg/dL), the highest quartile ( > 61.3 mg/dL) was associated with a 49% higher risk of myocardial infarction and a 28% higher risk of all-cause mortality, with these associations remaining significant after adjustment for traditional risk factors. The German Chronic Kidney Disease (GCKD) study (47) enrolled 5,043 patients with mild-to-severe CKD and found that each 10 mg/dL increase in Lp(a) concentration was associated with a 6.5% increase in CVD risk, and patients with Lp(a) ≥ 70 mg/dL had a 77.5% higher cardiovascular risk compared with those with Lp(a) < 30 mg/dL. In our nomogram, Lp(a) contributed a Δ*χ^2^* of 7.354 (*P* = 0.007) and a ΔC-index of 0.038 (95% CI 0.024–0.111, *P* = 0.033) (Supplementary Table S5a). Its NRI and IDI were consistent across time points [e.g., 24-month NRI: 0.213 (95% CI 0.111–0.488), 36-month NRI: 0.150 (95% CI 0.005–0.433)], underscoring its stable, genetically determined contribution to residual cardiovascular risk beyond LDL-C (Supplementary Table S5b). As a genetically driven and stable biomarker, Lp(a) extends the risk assessment window to 20–30 years (48), revealing residual risk undetectable by conventional factors, and is particularly valuable for long-term risk stratification in CKD patients and for early identification of genetically susceptible individuals.

FER, identified as an important risk predictor in our study, was associated with a 0.9% increase in the risk of composite endpoints for every 10 μg/L elevation in patients with CKD (HR = 1.009, 95% CI: 1.001–1.017, *P* = 0.030). As the core iron storage protein, maintains cellular iron homeostasis under physiological conditions by sequestering free iron to prevent Fenton reaction-generated reactive oxygen species (ROS) (49). However, in the setting of CKD-related microinflammation, oxidative stress, and iron metabolism dysregulation, FER releases free iron via NCOA4-mediated selective autophagy (ferritinophagy), which catalyzes lipid peroxidation, drives ferroptosis, and directly damages cardiac muscle and vascular endothelium (49–53). CKD-related microinflammation upregulates FER as an acute-phase protein, thereby disrupting its iron storage function (54). At the same time, inflammatory cytokines such as IL-6 and TNF-α directly induce FER expression in CKD patients, explaining the phenomenon of elevated FER levels during functional iron deficiency (54). This observation is consistent with the lack of correlation between hypochromic erythrocyte percentage and FER levels in CKD patients (*r* = −0.217, *p* = 0.130) (55). Although CKD patients present with both iron deficiency and iron overload, the characteristic oxidative stress environment (e.g., reduced GPX4 activity) enhances NCOA4-mediated ferritinophagy. Even when absolute iron stores are insufficient, abnormal FER degradation can still release free iron to catalyze lipid peroxidation (56–58). The French GAD-TRANS cohort (*n* = 3,033) confirmed that this pathway drives CVD independently of total iron levels (58). Incorporating FER into the model helps identify the concealed “high-FER, normal-iron” high-risk phenotype in CKD, reflects the degree of autophagic activation, and guides safe iron supplementation strategies in patients with iron deficiency. In our nomogram, FER contributed a Δ*χ^2^* of 4.264 (P = 0.039) and a ΔC-index of 0.014 (95% CI 0.009–0.033, *P* = 0.048) (Supplementary Table S5a). Although its overall effect is modest, its NRI and IDI were most prominent at 36 months (NRI: 0.462 [95% CI 0.090–0.640]; IDI: 0.057 [95% CI 0.002–0.126]), suggesting that ferritin captures cumulative iron-mediated injury over time, which is particularly relevant in advanced CKD (Supplementary Table S5b).

EQ-5D is a standardized health-related quality of life (HRQL) assessment tool that generates a single health utility index ranging from 0 to 1 by measuring five dimensions mobility, self-care, usual activities, pain/discomfort, and anxiety/depression (59, 60). Its core value lies in transforming multidimensional health states into quantifiable utility values (61). Research confirms that EQ-5D effectively captures CVD severity and health outcomes. For example, lower EQ-5D scores show a significant correlation with angina severity (P < 0.001), and each increase in Canadian Cardiovascular Society (CCS) classification reduces the utility value by 0.11 (95% CI 0.09–0.13) (62). Importantly, our study further demonstrates significant CVD predictive value of EQ-5D score in CKD populations. In our nomogram, EQ-5D contributed a **Δ*χ^2^*** of 5.037 (*P* = 0.025) and a ΔC-index of 0.019 (95% CI 0.009–0.026, *P* = 0.041) (Supplementary Table S5a). Its time-dependent NRI and IDI were modest but consistently positive across time points [e.g., 24-month NRI: 0.085 (95% CI 0.020–0.366); 36-month NRI: 0.250 (95% CI 0.031–0.467)] (Supplementary Table S5b). Every 0.1-point increase in EQ-5D utility corresponds to a 22% lower CVD risk. This association likely stems from shared CKD-CVD pathophysiological mechanisms including systemic inflammation, endothelial dysfunction, and calcium-phosphate metabolism disorders (63). Specifically, renal insufficiency-induced anemia exacerbates angina while calcium-phosphate imbalances accelerate vascular calcification (64, 65). These pathways collectively amplify CVD’s impact on quality of life. Meanwhile, EQ-5D’s multidimensional structure quantifies such additive effects by integrating subjective health dimensions like pain and anxiety thereby enhancing model sensitivity to silent cardiovascular events (66). Previous studies (32, 62, 67–69) have predominantly focused on quality-of-life assessments within single-disease contexts. Our work establishes for the first time the value of EQ-5D in predicting CVD and mortality risk among CKD patients. These findings provide a basis for applying EQ-5D score as a cardiovascular risk management tool in this population.

Existing CVD risk prediction models rely primarily on traditional risk factors (e.g., age, sex, systolic blood pressure, total cholesterol, and smoking status). However, the impact of these factors differs significantly in patients with CKD compared to the general population. Some traditional risk factors such as hypertension elevated BMI and high cholesterol show no association with reduced survival in advanced CKD or dialysis patients sometimes even demonstrating paradoxical effects (70). This limits their applicability for CKD populations. Traditional models like the Framingham Risk Score and ASCVD Risk Score focus on atherosclerotic events but overlook CKD-specific pathophysiology leading to inadequate sensitivity for predicting adverse outcomes (71). The HFRS targets confirmed heart failure populations using indicators like LVEF and BNP making it unsuitable for early screening in CKD patients (72). While QRISK3 includes CKD staging it neglects psychological and quality-of-life dimensions and its complexity burdens primary care settings (73).

Our novel model introduces the EQ-5D score capturing bio-psychosocial interactions which addresses traditional models’ neglect of overall patient health. It also incorporates new biomarkers: GDF15 quantifies cardio-renal tissue stress, FER reflects inflammation and autophagy from iron metabolism dysregulation, and Lp(a) signals inherited lipid risk, enhancing risk stratification precision. Integrating these with EQ-5D provides multi-dimensional monitoring of metabolic dysfunction organ damage and social function decline while compensating for EQ-5D’s ceiling effect (74). Unlike models (72) requiring expensive tests like NT-proBNP or advanced imaging our approach uses routinely available blood tests for GDF15, Lp(a) and FER plus the rapid EQ-5D questionnaire eliminating barriers in resource-limited settings.

This model directly guides clinical decisions through risk-stratified management. Low-risk patients scoring under 40 maintain standard CKD care with regular EQ-5D monitoring to prevent psychological burden. Medium-risk scorers between 40 and 80 receive intensified lifestyle and metabolic interventions like dietary adjustments to lower Lp(a) or counseling to improve EQ-5D. High-risk patients above 80 prioritize cardio-renal protective therapies. Specifically it personalizes iron treatment for functional iron deficiency defined as transferrin saturation under 20 percent with inflammatory hyperferritinemia between 30 and 100 μg/L overcoming iron utilization barriers. For patients with controlled LDL-C but persistently elevated Lp(a) RNA-targeted therapies address residual risk filling gaps in conventional lipid management.

Methodologically, this study adopted a hybrid analytical strategy integrating LASSO regularization, ensemble learning (XGBoost/RF) and traditional Cox regression, effectively addressing the limitations of single-model approaches. The machine learning components precisely captured associations between FER, Lp(a), EQ-5D and cardiovascular outcomes, while the Cox model ensured clinical interpretability through hazard ratio quantification. The implementation of a dynamic nomogram further enhanced clinical utility by providing time-stratified risk projections, thereby resolving temporal heterogeneity in cardiovascular pathophysiology. This integrated methodology establishes a novel paradigm for precision prognostics in multimorbid populations, balancing computational sophistication with actionable clinical insights.

Despite the strengths of our study, there are several limitations to note. Firstly, the single-center design and moderate sample size (*n* = 440) may limit the generalizability of findings, particularly the applicability of peritoneal dialysis-specific risk profiles. Second, although we employed a multi-stage feature selection process (univariate Cox screening, LASSO, RF, and XGBoost) to identify robust predictors, the relatively limited number of events (*n* = 104) raises the possibility of model instability and overfitting. Internal validation via bootstrap (1,000 resamples) mitigated this concern to some extent, but external validation in independent cohorts remains essential to confirm the model’s stability and generalizability. Third, our cohort included patients with CKD stages G3a-G5, of whom 386 (87.7%) had advanced disease (stages G4-G5), and 57.27% were receiving peritoneal dialysis. While this composition reflects a high-risk population in whom HF risk stratification is most clinically relevant, the model’s applicability to earlier CKD stages (e.g., G1-G2 or even G3a with milder disease) remains uncertain. Moreover, the number of endpoint events in CKD G3 and G4 stages was limited, which prevented us from performing formal stratified analyses for each individual CKD stage. Therefore, external validation in cohorts enriched for early stage CKD is needed before extrapolating our findings to that population. Fourth, the median follow-up of 29 months is insufficient to evaluate long-term (>5 years) predictors. Fifth, our primary endpoint was a composite of incident heart failure and all-cause death (including non-cardiovascular death). Ideally, we would have analyzed these components separately to clarify whether each predictor is primarily associated with HF development or reflects general mortality risk. However, the sample size and event counts (62 incident HF events, 42 all-cause deaths) limited the statistical power for such component-specific Cox models; therefore, we did not perform separate modeling. This should be considered when interpreting the results, as the predictors may contribute differently to each component. Sixth, external validation in multinational cohorts is required before clinical implementation. Nevertheless, our developed dynamic online tool (https://jiang221.shinyapps.io/dynnomapp) already holds immediate translational value, enabling real-time risk stratification in nephrology clinics.

## 5 Conclusion

In conclusion, this study developed a novel dynamic nomogram that accurately stratifies the risk of incident HF and all-cause mortality in patients with CKD. By integrating multidimensional predictors—age, GDF15, FER, Lp(a), and EQ-5D score—this tool offers clinicians with a practical, biomarker-enhanced framework for early risk identification.

## Data Availability

The datasets presented in this article are not readily available because the dataset is available from the corresponding author, Doc. Ying Wang, with permission from the First Affiliated Hospital of Anhui Medical University and upon justifiable request. Requests to access the datasets should be directed to Ying Wang, ying.wang@ahmu.edu.cn.
