# Supplementary material for: Development of a nomogram for predicting incident heart failure and all-cause mortality in patients with chronic kidney disease: a 3-year follow-up study
Source: Front Med (Lausanne). 2026 May 8;13:1784717. doi: 10.3389/fmed.2026.1784717 (PMC13194158; doi:10.3389/fmed.2026.1784717)
Supplement: Supplementary file 1 [file Data_Sheet_1.docx]

**Supplementary Table S1.** Distribution of CKD stages and composite endpoint events (incident heart failure and all-cause mortality) in the study cohort.

| **CKD Stage** | **Total Enrolled Patients [n (%)]** | **Composite Endpoint Events [n (%)]** | **Event Incidence Rate** |
| --- | --- | --- | --- |
| G3 | 54 (12.3%) | 4 (3.8%) | 7.4% |
| G4 | 28 (6.4%) | 10 (9.6%) | 35.7% |
| G5 (ESKD) | 358 (81.4%) | 90 (86.5%) | 25.1% |
| **Total** | **440 (100.0%)** | **104 (100.0%)** | **23.6%** |

**Supplementary Figure S1.** Schoenfeld residual plots for testing the proportional hazards assumption.


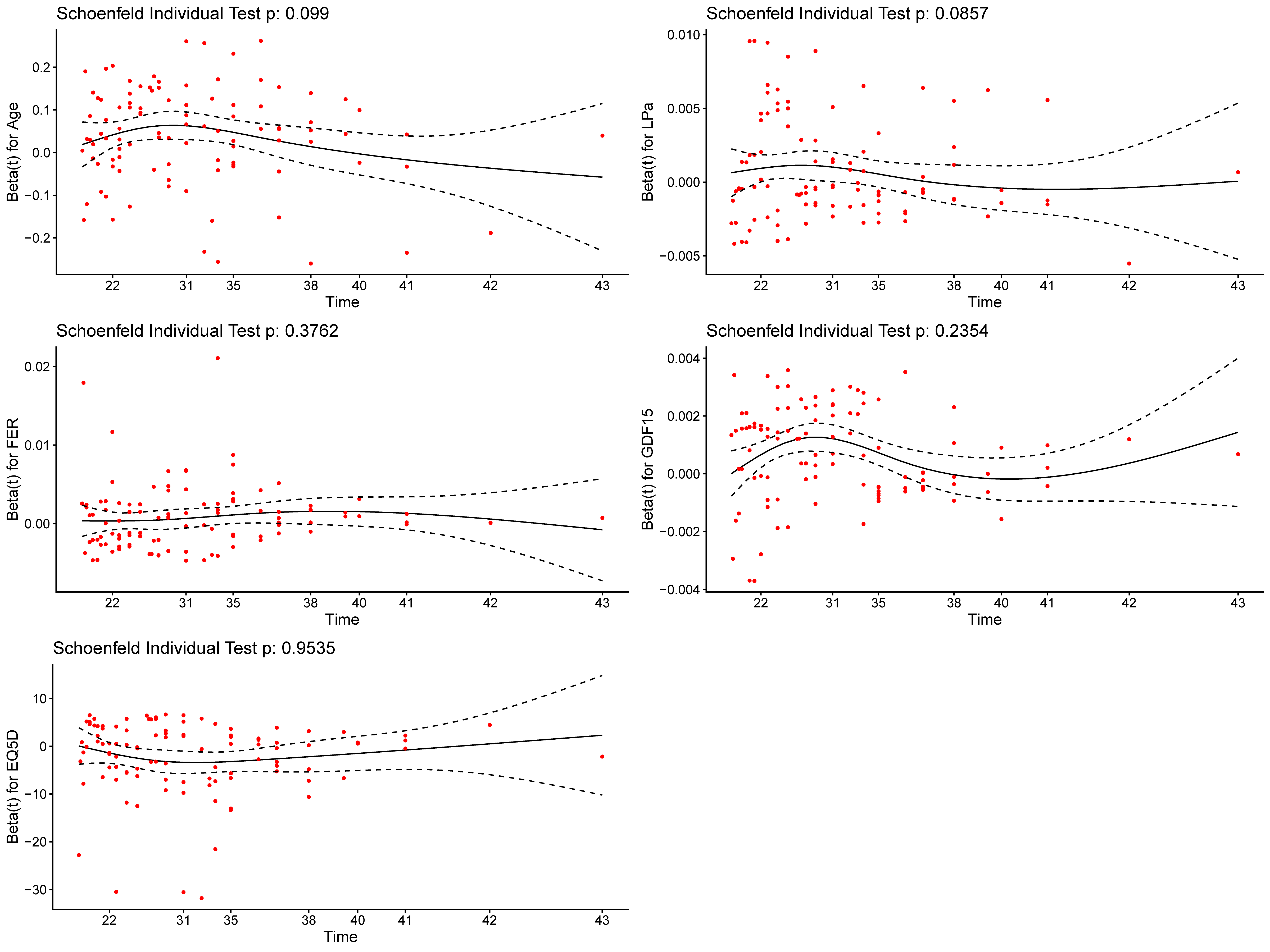


**Supplementary Table S2.** Collinearity diagnostics (VIF) for the final predictors.

| **Variable** | **VIF** |
| --- | --- |
| Age | 1.008244 |
| LPa | 1.014285 |
| FER | 1.048522 |
| GDF15 | 1.005330 |
| EQ5D | 1.045680 |

**Supplementary Table S3.** Multivariable Cox regression models for composite endpoint with and without CKD stage

|  | **Model 1** | | **Model 2** | |
| --- | --- | --- | --- | --- |
|  | **HR (95%CI)** | **P** | **HR (95%CI)** | **P** |
| **GDF15** | 1.076 (1.037–1.118) | **< 0.001** | 1.089 (1.043–1.138) | **< 0.001** |
| **FER** | 1.009 (1.001–1.017) | **0.030** | 1.010 (1.001–1.019) | **0.022** |
| **EQ-5D** | HR = 0.099 (0.013–0.755) | **0.026** | 0.089 (0.011–0.701) | **0.022** |
| **Lp(a)** | 1.011 (1.004–1.018) | **0.002** | 1.011 (1.003–1.019) | **0.011** |
| **Age** | 1.030 (1.006–1.056) | **0.016** | 1.034 (1.004–1.064) | **0.026** |
| **CKD stage**  **(per stage increase)** |  |  | 1.428 (0.730–2.793) | 0.298 |

**Supplementary Table S4.** Calibration slopes and Brier scores of the nomogram at 24, 27, 32, and 36 months in the training and validation cohorts

| **Time Point** | **24-month** | **27-month** | **32-month** | **36-month** |
| --- | --- | --- | --- | --- |
| **Training Cohorts** |  |  |  |  |
| Brier Scores | 0.060 | 0.073 | 0.102 | 0.160 |
| Calibration Slopes | 0.977 | 1.050 | 1.064 | 1.060 |
| **Validation Cohorts** |  |  |  |  |
| Brier Scores | 0.081 | 0.090 | 0.128 | 0.135 |
| Calibration Slopes | 0.811 | 0.947 | 0.854 | 0.864 |

**Supplementary Table S5a.** Overall incremental predictive value of each predictor assessed by likelihood ratio test and change in C-index

| **Variable** | **∆*χ²* (LRT)** | ***P*** | **∆C-index (95%CI)** | ***P*** |
| --- | --- | --- | --- | --- |
| **GDF15** | 14.701 | <0.001 | 0.074 (0.011-0.136) | <0.001 |
| **FER** | 4.264 | 0.039 | 0.014 (0.009-0.033) | 0.048 |
| **EQ-5D** | 5.037 | 0.025 | 0.019 (0.009-0.026) | 0.041 |
| **Lp(a)** | 7.354 | 0.007 | 0.038 (0.024-0.111) | 0.033 |
| **Age** | 6.966 | 0.008 | 0.041 (0.020-0.064) | 0.024 |

LRT:likelihood ratio test

**Supplementary Table S5b**. Time‑dependent net reclassification improvement (NRI) and integrated discrimination improvement (IDI) of each predictor at 24, 27, 32, and 36 months

| **Time** | **Metric** | **GDF15** | **FER** | **EQ-5D** | **Lp(a)** | **Age** |
| --- | --- | --- | --- | --- | --- | --- |
| 24-month | NRI (95% CI) | 0.262 (0.010 - 0.541) | 0.203 (0.118 - 0.493) | 0.085 (0.020 - 0.366) | 0.213 (0.111 - 0.488) | 0.230 (0.057 - 0.364) |
|  | IDI (95% CI) | 0.028 (0.017 - 0.092) | 0.015 (0.011 - 0.072) | 0.028 (0.005 - 0.042) | 0.027 (0.009 - 0.034) | 0.021 (0.004 - 0.093) |
| 27-month | NRI (95% CI) | 0.332 (0.073 - 0.549) | 0.129 (0.125 - 0.380) | 0.171 (0.092 - 0.420) | 0.332 (0.038 - 0.521) | 0.202 (0.150 - 0.405) |
|  | IDI (95% CI) | 0.076 (0.006 - 0.183) | 0.018 (0.004 - 0.042) | 0.015 (0.013 - 0.055) | 0.029 (0.008 - 0.099) | 0.036 (0.004 - 0.106) |
| 32-month | NRI (95% CI) | 0.361 (0.150 - 0.557) | 0.215 (0.011 - 0.452) | 0.119 (0.098- 0.317) | 0.212 (0.009 - 0.454) | 0.213 (0.195 - 0.415) |
|  | IDI (95% CI) | 0.117 (0.032 - 0.238) | 0.015 (0.013 - 0.058) | 0.030 (0.011 - 0.043) | 0.028 (0.014 - 0.095) | 0.040 (0.005 - 0.119) |
| 36-month | NRI (95% CI) | 0.451 (0.208 - 0.659) | 0.462 (0.090 - 0.640) | 0.250 (0.031 - 0.467) | 0.150 (0.005 - 0.433) | 0.287 (0.069 - 0.299) |
|  | IDI (95% CI) | 0.140 (0.050 - 0.265) | 0.057 (0.002 - 0.126) | 0.052 (0.006 - 0.116) | 0.029 (0.002 - 0.084) | 0.023 ( 0.009 - 0.070) |

NRI:net reclassification improvement;

IDI:integrated discrimination improvement
